# Mobilization and Cellular Distribution of Phosphate in the Diatom *Phaeodactylum tricornutum*

**DOI:** 10.3389/fpls.2020.00579

**Published:** 2020-06-03

**Authors:** Gianluca Dell’Aquila, Stefan Zauner, Thomas Heimerl, Jörg Kahnt, Vera Samel-Gondesen, Simon Runge, Franziska Hempel, Uwe G. Maier

**Affiliations:** ^1^Laboratory for Cell Biology, Philipps University of Marburg, Marburg, Germany; ^2^SYNMIKRO Research Center, Marburg, Germany; ^3^Max Planck Institute for Terrestrial Microbiology, Marburg, Germany

**Keywords:** phosphate, Phaeodactylum tricornutum, alkaline phosphatases, phosphate transporters, transcriptional regulation, phosphate homeostasis

## Abstract

Unicellular organisms that live in marine environments must cope with considerable fluctuations in the availability of inorganic phosphate (P_i_). Here, we investigated the extracellular P_i_ concentration-dependent expression, as well as the intracellular or extracellular localization, of phosphatases and phosphate transporters of the diatom *Phaeodactylum tricornutum*. We identified P_i_-regulated plasma membrane-localized, ER-localized, and secreted phosphatases, in addition to plasma membrane-localized, vacuolar membrane-localized, and plastid-surrounding membrane-localized phosphate transporters that were also regulated in a P_i_ concentration-dependent manner. These studies not only add further knowledge to already existing transcriptomic data, but also highlight the capacity of the diatom to distribute P_i_ intracellularly and to mobilize P_i_ from extracellular and intracellular resources.

## Introduction

Phosphorus (P) is an essential component of numerous biomolecules and most readily accessed by cells in the form of inorganic phosphate (P_i_). However, P_i_ uptake and intracellular distribution can be a challenging process for unicellular organisms, especially those living in an environment with fluctuating P_i_ concentrations. In the oceans, for example, the phosphate concentration is low when primary production is high (Conkright et al., 2000). In addition to dissolved inorganic phosphates (DIPs), other major phosphate sources include dissolved organic phosphates (DOPs) such as phosphate monoesters, phosphate diesters, and phosphonates (Yamaguchi et al., 2014; Whitney and Lomas, 2019). In the case of DOPs, P_i_ esters can be utilized as a P_i_ source through the activity of enzymes such as alkaline phosphatases and esterases (e.g., Whitney and Lomas, 2019).

Diatoms are important members of the phytoplankton (Armbrust, 2009). They are very successful in terms of biomass production and CO_2_ fixation and contribute greatly to global climate regulation (e.g., Benoiston et al., 2017). In terms of cell biology, diatoms are highly compartmentalized, and biomass production is dependent on their plastid, which is surrounded by four membranes (Maier et al., 2000). To cope with changing phosphate concentrations, diatoms have evolved several strategies for fine-tuning phosphate acquisition with metabolism, as evidenced by the results of recent transcriptomic and proteomic studies (Dyhrman et al., 2012; Yang et al., 2014; Shih et al., 2015; Cruz de Carvalho et al., 2016; Zhang et al., 2016; Alipanah et al., 2018). These data, although generated by different experimental designs (e.g., duration of P_i_ starvation) or diatom species, indicated a global transcriptomic reprogramming, which should influence the protein composition of many cell compartments, accompanied by several physiological responses. In addition, a Myb-like transcription factor, P starvation response (PtPSR), which controls cellular adaptations to P_i_ stress, has recently been identified in *Phaeodactylum tricornutum* (Sharma et al., 2019). The response to P_i_ limitation correlates with a specific transcriptional upregulation of genes coding for alkaline phosphatases that are necessary for phosphate acquisition from DOP. Additionally, *P. tricornutum* has been reported to secrete the alkaline phosphatase PhoA (Lin et al., 2013; Buhmann et al., 2016; Erdene-Ochir et al., 2019), as well as a phytase-like protein (Buhmann et al., 2016; Erdene-Ochir et al., 2019), into the culture medium. A phosphate-specific reprogramming can be identified by P_i_ transporters as well, as evidenced by the transcriptional upregulation of sodium-dependent phosphate cotransporters, permeases, and hydrogen–phosphate cotransporters (Dyhrman et al., 2012; Yang et al., 2014; Cruz de Carvalho et al., 2016; Alipanah et al., 2018) in P_i_-depleted media.

In this study, we investigated P_i_ mobilization, cellular P_i_ uptake, and intracellular P_i_ distribution in the diatom *P. tricornutum*. We first analyzed the culture medium for the presence of secreted phosphatases. Next, we screened genomic data (Bowler et al., 2008; Rastogi et al., 2018) for phosphatases and phosphate transporters and investigated whether their expression was regulated in a phosphate-dependent manner. The P_i_-regulated candidates were further investigated according to their *in vivo* localization, which resulted in the detection of extracellular or intracellular proteins. Together with known expression data, our findings provide a comprehensive insight into the complex and balanced P_i_ metabolism related to phosphatases and phosphate transporters of diatoms.

## Materials and Methods

### *In Silico* Analysis

Putative factors involved in P_i_ homeostasis were identified using available transcriptomic data (Yang et al., 2014; Cruz de Carvalho et al., 2016; Alipanah et al., 2018). Additionally, the “Phatr2_domaininfo_FilteredModels2.tab” file^1^ was screened for protein domains known to be required for P_i_ homeostasis (e.g., PF02690 for the Na^+^/P_i_ cotransporters, SPX domain, H^+^-PPase). The identified proteins were then used as bait for local BLAST analyses in the Phatr2 and Phatr3 databases (Bowler et al., 2008; Rastogi et al., 2018)^2^ ^,3^ using default settings. N-terminal signal peptides of the analyzed proteins were predicted using SignalP3.0^4^ and SignalP4.1^5^. For transmembrane helix prediction, several web-based tools were utilized, namely, TOPCONS^6^, Phobius^7^, TMHMM^8^, and TMpred^9^. Conserved domains were determined using the NCBI Conserved Domain Database^10^. Protein mass and theoretical isoelectric point estimation were determined using PEPTIDEMASS (Wilkins et al., 1997)^11^. Analysis of putative phosphorylation sites was performed using DISPHOS 1.3^12^.

### Vector Construction

For localization studies, genes were amplified from *P. tricornutum* (strain UTEX646) genomic DNA or cDNA using Q5 5× Master Mix (New England BioLabs, Ipswich, MA, United States) and gene-specific primers synthesized by Sigma-Aldrich, St. Louis, MO, United States. Notably, the sequences obtained were slightly different to that of *P. tricornutum* CCAP1055/1^13^. Cloning was performed using either restriction sites or Gibson assembly (Gibson et al., 2009). For cloning using restriction enzymes, primers that contained terminal restriction sites were used, and the amplicons for *PtPhos3*, *PtPhos5*, *PtPhos6*, *PtPho4*, *PtNap_i_1*, *PtNap_i_2*, *PtNap_i_4*, *PtNap_i_5*, *PtVpt1*, *PtVtc1*, *PtVtc3*, and *PtVtc4* genes were cloned into pJet1.2/blunt using the Clone Jet PCR Cloning Kit (Thermo Fisher Scientific, Waltham, MA, United States). After sequencing, inserts were cloned into the shuttle vector pPha-NR (GenBank: JN180663) upstream of eGFP. *PtPhos6* (eGFP downstream of the gene), *PtPhos7*, *PtPhos8*, *PtHp_i_1*, and *PtNap_i_3* eGFP-fusion protein constructs were generated *via* Gibson assembly. Primers sequences are available in Supplementary Table S1 (Supplementary File S1). For analysis of transcriptional regulation, Gibson assembly was used to generate GFP cassettes with different promoter/terminator units in the pPha-T1 vector (GenBank: AF219942). For each investigated gene, at least 900 bp upstream and 485 bp downstream of the coding sequence were used, including untranslated regions if present. Sequences and region lengths are included in Supplementary File S2.

### Culture Conditions and Biolistic Transformation

*Phaeodactylum tricornutum* (Bohlin, UTEX646) was cultivated in f/2 medium without silica (Guillard, 1975) containing 1.66% (wt/vol) Tropic Marin (Dr. Biener GmbH, Wartenberg, Germany) and 2 mM Tris–HCl (pH 8.0) under constant light (8,000–10,000 lx) and shaking (100–150 revolutions/min) or on plates with solid agar-containing (1.3% w/vol) f/2 medium at 21°C. Transformation of *P. tricornutum* was carried out as previously described (Apt et al., 1996). Transformants were selected on f/2-agar plates supplemented with zeocin (InvivoGen, San Diego, CA, United States) at a final concentration of 75 μg/mL. For transcriptional regulation and enzyme-labeled fluorescence (ELF) experiments, *P. tricornutum* cells were maintained in the exponential growth phase for 7 days in standard f/2 medium supplemented with 36 μM NaH_2_PO_4_. Before experimental treatment, approximately 1 × 10^8^ cells were harvested (1,500 × g, 21°C, 10 min), washed twice with P_i_-free f/2 medium, and transferred into 100-mL Erlenmeyer flasks containing 50 ml (initial cell concentration 2 × 10^6^ cells/mL) of f/2 medium with 0, 36, 72, 90, and 108 μM P_i_ (NaH_2_PO_4_) and incubated for 2 days before protein and microscopy analysis. Cell numbers were determined using a Thoma counting chamber (Hecht Assistent, Sondheim vor der Rhön, Bayern, Germany).

### Protein Isolation, Sodium Dodecyl Sulfate–Polyacrylamide Gel Electrophoresis, and Western Blot Analysis

For protein isolation, approximately 1 × 10^8^ cells in the exponential growth phase were harvested (1,500 × *g*, 21°C, 10 min) and resuspended in lysis buffer (1.85 mM NaOH and 7.5% [vol/vol] β-mercaptoethanol). Proteins present in the medium were processed as described in Hempel and Maier (2012) by harvesting proteins in Amicon Ultra Centrifugal Filters (10-kDa cutoff) (Merck, Darmstadt, Germany) after filtration (0.2-μm filters). Protein precipitation was performed using trichloroacetic acid (10% vol/vol), and the protein pellet was washed at least three times with 80% acetone. For each sample, 15 to 20 μg of protein were loaded on a 12.5% sodium dodecyl sulfate–polyacrylamide gel electrophoresis (SDS–PAGE) gel and separated. In the secretion experiment, the gel was stained with Instant Blue (Expedeon, Heidelberg, Germany) Coomassie protein stain following the manufacturer’s instructions. For Western blot, proteins were transferred to a nitrocellulose membrane using a Pierce Semi-Dry Blotter (Thermo Fisher Scientific). Immunodetection was performed with antibodies against EGFP (1:3,000; Rockland, Pottstown, PA, United States) using α-tubulin (1:2000; Sigma-Aldrich) as loading control. Signal detection was performed by cutting the membranes at 40 kDa in order to use the upper part for the α-tubulin at the lower part for the eGFP antibodies.

### Identification of Secreted Phosphatases

Two prominent bands for proteins obtained from the medium were initially identified *via* matrix-assisted laser desorption/ionization–time-of-flight (MALDI–TOF) analysis. Excised gel slices were cut into small pieces, destained with 30% isopropyl alcohol containing 60 mM ammonium carbonate and 30 mM thioglycolic acid, dehydrated with isopropyl alcohol, and dried. The gel pieces were then rehydrated in 10% acetonitrile containing 5 mM ammonium bicarbonate, 8 mM DTT, and 2.5 μg/mL sequencing-grade modified trypsin (Promega, Madison, WA, United States) and incubated overnight at 30°C. The resulting peptide solution was spotted together with matrix solution (α-cyano-4-hydroxycinnamic acid) on a MALDI plate. Automated MALDI–TOF–TOF analysis was carried out on a 4800 Proteomics Analyzer (AB Sciex, Darmstadt, Germany) in positive ion reflector mode and externally calibrated. Mass spectrometric data were searched against an in-house protein database using Mascot embedded into the GPS explorer software (AB Sciex). The obtained data were reproduced by a different method. After destaining, samples were reduced, carbamidomethylated, and digested “in-gel” by the addition of sequencing-grade modified trypsin (Serva, Heidelberg, Germany) and incubated at 37°C overnight. Peptides were desalted and concentrated using Chromabond C18WP spin columns (Macherey-Nagel, Düren, Germany, part no. 730522). Finally, peptides were dissolved in 25 μL of water with 5% acetonitrile and 0.1% formic acid. The mass spectrometric analysis of the samples was performed using an Orbitrap Velos Pro mass spectrometer (Thermo Fisher Scientific). An Ultimate nanoRSLC-HPLC system (Dionex, Sunnyvale, CA, United States) equipped with a custom end-fritted 50-cm × 75-μm C18 RP column filled with 2.4-μm beads (Dr. Maisch, Ammerbuch, Germany) was connected online to the mass spectrometer through a Proxeon (Odense, Denmark) nanospray source. Approximately 1–15 μL (depending on peptide concentration and sample complexity) of the tryptic digest was injected onto a 300-μm ID × 1-cm C18 PepMap preconcentration column (Thermo Fisher Scientific). Automated trapping and desalting of the sample were performed at a flow rate of 6 μL/min using water/0.05% formic acid as the solvent. Separation of the tryptic peptides was achieved with the following gradient of water/0.05% formic acid (solvent A) and 80% acetonitrile/0.045% formic acid (solvent B) at a flow rate of 300 nL/min: holding 4% B for 5 min, followed by a linear gradient to 45% B within 30 min and a linear increase to 95% solvent B in an additional 5 min. The column was connected to a stainless steel nanoemitter (Proxeon), and the eluent was sprayed directly onto the heated capillary of the mass spectrometer using an electrospray potential of 2300 V. A survey scan with a resolution of 60,000 within the Orbitrap mass analyzer was combined with at least three data-dependent MS/MS scans with dynamic exclusion for 30 s using either CID with the linear ion trap or HCD combined with Orbitrap (Thermo Fisher Scientific, Waltham, MA, United States) detection at a resolution of 7,500. Data analysis was performed using Proteome Discoverer 2.2 (Thermo Fisher Scientific) with the SEQUEST search engine. The mass spectrometric proteomic data were deposited to the ProteomeXchange Consortium *via* the PRIDE (Perez-Riverol et al., 2019) partner repository with the dataset identifiers PXD016456 and 10.6019/PXD016456.

### Confocal Laser Scanning Microscopy

To analyze the *in vivo* localization of each eGFP fusion protein, gene expression was induced by incubating the cells in f/2 medium containing 0.9 nM NaNO_3_ instead of 1.5 nM NH_4_Cl for 24 h in sterile reaction tubes under the conditions described above. Localization of GFP fusion proteins was performed using a Leica TCS SP2 confocal laser scanning microscope (Leica, Wetzlar, Germany) with an HCXPL APO40/1.25-0.75 Oil CS objective. Excitation of eGFP and plastid autofluorescence was performed at 488 nm using a 65-mW argon laser. For eGFP, emission was detected at a bandwidth of 500–520 nm and autofluorescence at 625–720 nm.

### Enzyme-Labeled Fluorescence

The ELF assay was performed using a modified protocol from González-Gil et al. (1998). A 1-mL sample of a P_i_-depleted or P_i_-replete [36, 72, and 108 μM P_i_ (NaH_2_PO_4_)] wild-type culture (see above) was harvested (1,500 × *g*, 21°C, 10 min), resuspended in 95 μL of f/2 medium (different P_i_ concentrations) containing 5 μL of ELF-97 (Endogenous Phosphatase Detection Kit; Molecular Probes, Eugene, OR, United States), and incubated in the dark at room temperature. As a control, cells were incubated with ELF buffer only. After 30 min of incubation, the cells were washed with f/2 medium containing the respective P_i_ concentration. ELF-97 fluorescence was detected using a Zeiss Axioplan2 (Carl Zeiss Microscopy GmbH, Oberkochen, Germany) equipped with a DAPI filter (Zeiss filter set 01, excitation BPP 365/12, beam splitter FT 395, emission LP 397).

### Electron Microscopy

Precultures were grown in f/2 medium, washed three times with P_i_-free f/2 medium, and then used to inoculate 50-mL flasks containing either P_i_-free f/2 medium or standard f/2 medium as described above. After 2 days, cells were harvested by centrifuging aliquots of the cultures (1,500 × *g*, 10 min). Concentrated cells were then high-pressure frozen (HPF Compact 02; Wohlwend, CH, Sennwald, Switzerland); freeze-substituted in acetone containing 0.25% osmium tetroxide, 0.2% uranyl acetate, and 5% water (0.05% ruthenium red was added for the PtVtc3-eGFP samples) (AFS2; Leica); and embedded in Epon 812 substitute resin (Fluka Chemical, Buchs, Switzerland). Freeze-substitution was performed as follows: −90°C for 20 h, from −90°C to −60°C for 1 h, −60°C for 8 h, −60°C to −30°C for 1 h, −30°C for 8 h, and −30°C to 0°C for 1 h. At 0°C, the samples were washed three times with acetone before a 1:1 mixture of Epon 812 substitute resin and acetone was applied at room temperature. After 2 h, the 1:1 mixture was substituted with pure resin and left overnight. After another substitution with fresh Epon, the samples were polymerized at 60°C for 2 days. Sections (50 nm) were cut from the Epon blocks (UC7, Leica; DiAtome, Biel, Switzerland) and transferred to 100-mesh copper grids coated with pioloform. Immunolabeling of Vtc3-eGFP samples was carried out as recently described (Liu X. et al., 2016). Samples were analyzed using a JEOL JEM2100 transmission electron microscope equipped with a fast-scan 2k CCD camera (F214; TVIPS, Gauting, Germany).

## Results

### Extracellular Phosphate Acquisition

**FIGURE 1:**
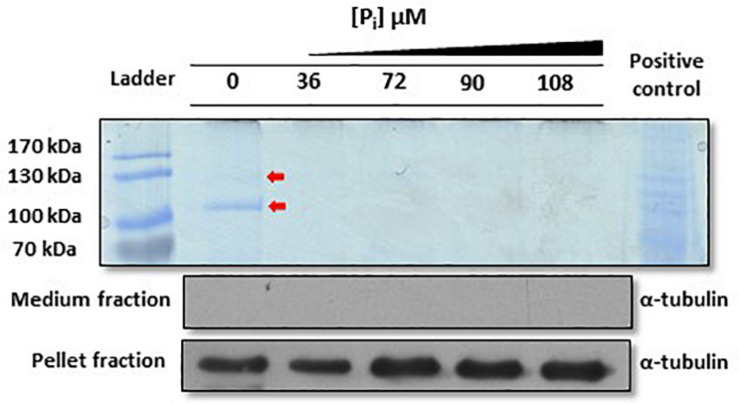
Analysis of *Phaeodactylum tricornutum* proteins secreted in F/2 medium under different inorganic phosphate (P_i_) concentrations. Coomassie blue staining revealed that two signals at approximately 130 and 100 kDa (red arrows, PtPhos2 and PtPhos1, respectively) are present only under P_i_-depleted conditions. A *P. tricornutum* cell lysate was used as positive control for the Coomassie staining. To verify that the protein extracts of the medium were not contaminated with lysed cells or cell debris, an aliquot of each sample was tested *via* Western blot for detection of α-tubulin. The same approach was performed with the protein extracts from the cell pellet (same blot) that served as loading control as well as an α-tubulin positive control. Scans of the entire blots and stained gels are available in Supplementary Figure S3.

One strategy to locally enrich the environment with P_i_ is to secrete enzymes that hydrolyze P_i–_containing molecules present in the ocean. While analyzing the secretion of monoclonal antibodies into the medium by the diatom *P. tricornutum* (Hempel and Maier, 2012), in addition to the heavy and light chains of the antibody, we detected two protein bands in Coomassie-stained protein gels (Figure 1). Both were analyzed *via* mass spectrometry and gave hits for two proteins (IDs: 49678 and 47612), which we called PtPhos1 and PtPhos2 (Supplementary Table S2). PtPhos1 is a secreted phosphatase already described and named PtAPase by Lin et al. (2013), whereas PtPhos2, predicted to be a phytase-like enzyme based on its domain structure, was first identified by Buhmann et al. (2016) as a secreted protein. *PtPhos1* and *PtPhos2* encode proteins containing a predicted signal peptide and, as expected, no transmembrane domain. Interestingly, the molecular masses indicated by Coomassie staining of SDS gels (PtPhos1: ∼100 kDa; PtPhos2: ∼130 kDa) differed significantly from those calculated based on their amino acid composition (PtPhos1: 77.2 kDa; PtPhos2: 83.8 kDa) (Figure 1). However, both proteins were identified by mass spectrometry and also showed an aberrant, high-mass band in Western blots when *PtPhos1* cDNA was expressed with a FLAG tag (data not shown). The secretion of these two phosphatases was confirmed by incubating the cells in medium containing different P_i_ concentrations. Coomassie staining showed that the phosphatases were secreted in P_i_-depleted medium (Figure 1). To further characterize the expression of *PtPhos1* and *PtPhos2* with respect to phosphate concentration, we cloned approximately 1,000 bp upstream and 500 bp downstream (promoter and terminator regions) of the coding sequence of both genes into the expression vector pPha-T1 (Zaslavskaia et al., 2000), such that the regulatory promoter and terminator regions could drive eGFP expression in the cytosol of the diatom (for the exact length of the promoter and terminator regions used here and in the following constructs, see Supplementary File S2).

**FIGURE 2:**
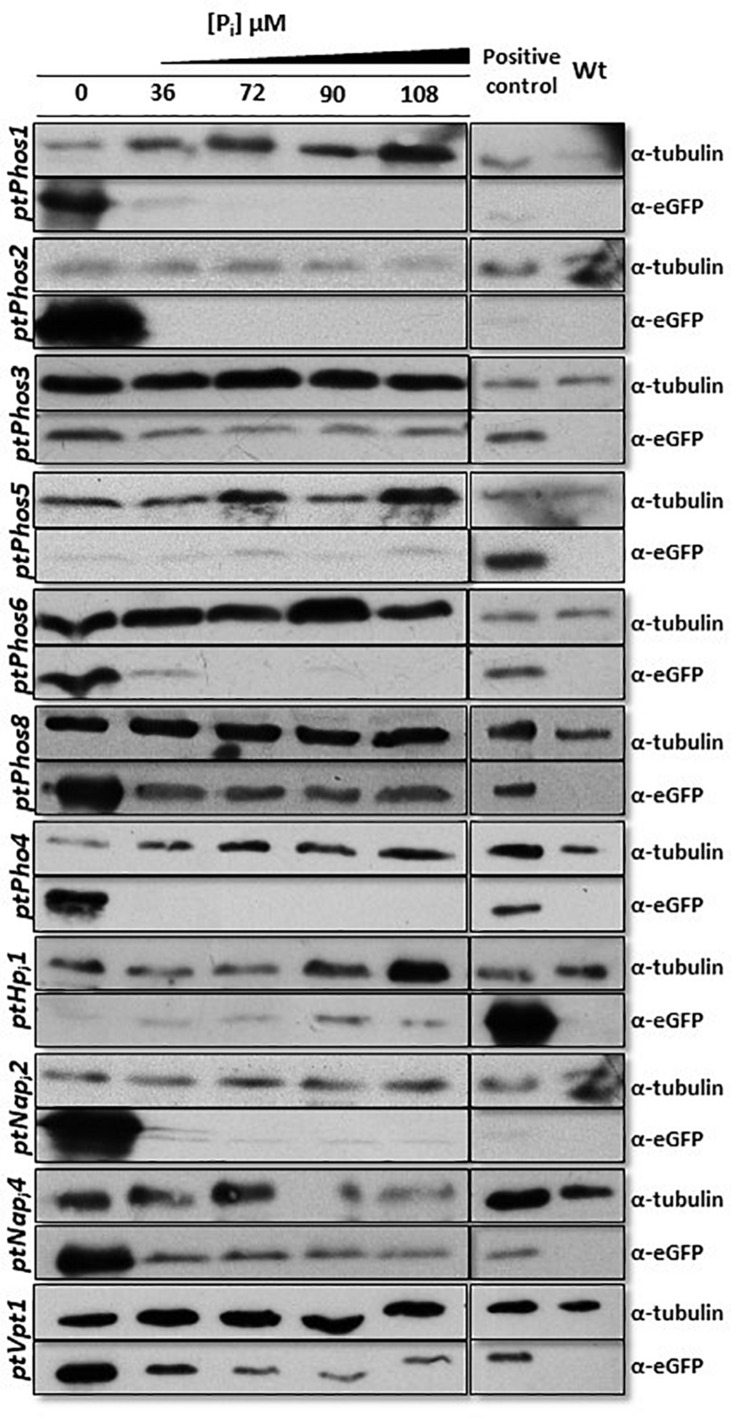
Western blot detection of eGFP protein fused with promoter/terminator cassettes from different phosphatases and P_i_ transporters. Strains expressing eGFP driven by different promoter/terminator cassettes (denoted on the left) were incubated under different P_i_ concentrations for 48 h. An anti–α-tubulin antibody was used as loading control. An eGFP-expressing strain (BTS/eGFP) was used to isolate eGFP and served as positive control. Wild-type protein extract was used as negative control for the eGFP antibody. A cropped section of the blots is shown. PtPhos3 and PtPhos6 were blotted together and shared the positive and negative controls, likewise PtPhos2 and PtNap_i_2. Scans of the entire blots are available in Supplementary Figure S3.

After transfection, the obtained transformants were incubated for 48 h in media containing different P_i_ concentrations (0, 36, 72, 90, and 108 μM). As the observed eGFP fluorescence was very weak in some cases (Supplementary Figure S1), we additionally determined the expression of the eGFP protein in individual clones by Western blot, leading to more precise results regarding the P_i_-controlled eGFP expression (Figure 2). Our results showed that the promoter/terminator regions of *PtPhos1* induced the P_i_-dependent expression of eGFP, with a peak under P_i_-limited conditions and basal expression under P_i_-replete conditions (36 and 72 μM). In contrast, the promoter/terminator regions of *PtPhos2* induced P_i_-regulated eGFP expression (Figure 2), but with no basal expression under P_i_-replete conditions.

### Regulation of PtPhos1 *via* Posttranslational Modifications

Our data indicated that *PtPhos1* was expressed at basal levels in P_i_-replete medium (Figure 2), whereas secretion patterns showed that PtPhos1 was not released under P_i_-replete conditions (Figure 1, ∼100-kDa band, red arrow). However, as secretion of this enzyme under P_i_-replete conditions would be expected to be costly for a cell when enzyme function is not essential, a further level of secretion regulation might be present. Interestingly, we observed that the protein sequence of the secreted PtPhos1 enzyme contains several putative phosphorylation sites after the signal peptide (Supplementary Figure S2), in contrast to that observed for PtPhos2. To determine whether reversible phosphorylation affects the secretion of the protein, we followed a phospho-mimicry approach. We expressed constructs in which serine residues in the N-terminus of PtPhos1, which might be important for phosphorylation, were converted either to glutamic acid (phospho-mimicry) or alanine, such no phosphorylation can occur in the latter. After the expression of the constructs, which additionally harbored a 1× FLAG tag at their C-terminus, we used Western blot to determine whether intracellular and secreted versions of PtPhos1 were present in the medium and in cell extracts. However, no sequence mutation-based effects were observed (data not shown).

### Expression of Additional Alkaline Phosphatases

We investigated six additional phosphatases (PtPhos3 to PtPhos8) (Table 1). PtPhos5 and PtPhos8 are encoded as putative preproteins with an N-terminal signal peptide. In addition, PtPhos3, PtPhos5, PtPhos6, PtPhos7, and PtPhos8 are predicted to be membrane-integrated phosphatases containing one membrane-spanning helix. Interestingly, for PtPhos5, PtPhos6, PtPhos7, and PtPhos8, the membrane-spanning region is predicted to be located at the ultimate C-terminus, while the catalytic regions of these alkaline phosphatases are predicted to be differentially localized. While PtPhos5 and PtPhos8 that contain a signal peptide would presumably act either in the lumen of a compartment or, when integrated into the plasma membrane, in the extracellular region, the catalytic domain of PtPhos6 and PtPhos7 might be exposed to the cytoplasm. The latter prediction is based on the fact that these enzymes might have a C-tail membrane anchor, which could be integrated into a membrane either spontaneously or *via* a guided entry (e.g., Borgese and Fasana, 2011). Notably, it remained unclear whether PtPhos4 contains a signal peptide. Additionally, this phosphatase does not have a membrane-spanning helix and could not be characterized as either intracellular or extracellular.

**TABLE 1:** Identified P_i_-regulated/non-regulated candidate proteins.

	Running name	ID	SP	THMs	Conserved domain	Transcriptional regulation
						Alipanah et al., 2018 (2 days–P_i_). Log_2_ fold change
Alkaline	PtPhos1	49678	+	−	PhoA	8.4
phosphatases	PtPhos2	47612	+	−	Phytase-like	7.4
	PtPhos3	45959	−	1 (N-term.)	MPP-PhoD	1.3
	PtPhos4	39432	/	−	PhoD	9.2
	PtPhos5	48970	+	1 (C-term.)	MPP-PhoD	/
	PtPhos6	45757	−	1 (C-term.)	MPP-PhoD	7.9
	PtPhos7	45174	−	1 (C-term.)	MPP-PhoD	/
	PtPhos8	47869	+	1 (C-term.)	Aty-PhoA	9.1
						Alipanah et al., 2018 (2 days–P_i_). Log_2_ fold change
Phosphate	PtPho4	23830	+	8	P_i_ permease	4.3
transporters	PtHp_i_1	17265	−	12	H^+^/P_i_ transporter	0.4
	PtHp_i_2	Bd462	−	10	H^+^/P_i_ transporter	/
	PtNap_i_1	33266	−	10	Na/P_i_ transporter	/
	PtNap_i_2	40433	−	10	Na/P_i_ transporter	5.8
	PtNap_i_3	47239	−	10	Na/P_i_ transporter	5.9
	PtNap_i_4	47667	−	10	Na/P_i_ transporter	3.7
	PtNap_i_5	49842	−	10	Na/P_i_ transporter	0.3
	PtNap_i_6	47666	−	8	Na/P_i_ transporter	4.6
	PtVpt1	19586	−	11	MFS_1 Sup. Fam. + SPX	4.3
						Alipanah et al., 2018 (2 days–P_i_). Log_2_ fold change
Vtc subunits	PtVtc1	48811	−	3	DUF202	/
	PtVtc2	35739	−	3	DUF202	1.8
	PtVtc3	48538	−	3	CYTH-like Pase sup. fam. + DUF202	/
	PtVtc4	50019	−	3	SPX + CYTH-like_Pase sup. fam.	−0.9

*Protein IDs refer to Phatr2 database (https://mycocosm.jgi.doe.gov/Phatr2/Phatr2.home.html). Notice that the ID of PtVtc1 is present only in the “all models” of Phatr2 database. (+), signal peptide predictions with significant score; (−), no signal peptide predicted; (C-term./N-term.), indicates the position of the predicted transmembrane helix; reported transcriptomic data are from Alipanah et al. (2018) (log_2_ fold change), determined after P_i_-starvation for 48 h in exponential phase, equivalent to the experiments of this work. (/), not determined.*

The expression data of Alipanah et al. (2018) indicated that *PtPhos6* and *PtPhos8* exhibit significant transcriptional upregulation under P_i_-depleted conditions. To investigate the effect of the promoter/terminator regions on phosphate-dependent protein expression, we investigated these regions of both phosphatases in more detail. As with *PtPhos1* and *PtPhos2*, we first fused the upstream and downstream regions of these phosphatase genes with eGFP such that eGFP was expressed in the cytoplasm. As again the observed eGFP fluorescence was very weak in some cases, we evaluated eGFP protein expression by Western blot analyses. Additionally, we determined the expression of *PtPhos3* [not or only moderately regulated according to the transcriptional data of Yang et al. (2014) and Alipanah et al. (2018)]. The protein expression driven by regulatory regions of PtPhos3 showed only a slight increase in P_i_-depleted media when compared with that under replete conditions (Figure 2). Testing of the promoter/terminator regions of *PtPhos8* indicated that they induced an increase in the expression of eGFP under P_i_-depleted conditions, whereas only a basal level of expression was detected under P_i_-replete conditions. The regulatory regions of the *PtPhos6* gene engendered the repression of EGFP expression in cells grown in medium containing double or more the amount of phosphate (72, 90, and 108 μM) (Figure 2). The transcriptional data (Table 1) indicated that the expression of *PtPhos5* and *PtPhos7* was not significantly, if at all, P_i_-dependent. We tested P_i_-regulated expression in case of *PtPhos5* exemplarily, using the expression module of the promoter/terminator region. In agreement with the transcriptional data, *PtPhos5* was constitutively expressed in our experiments (Figure 2).

### Localization of the Phosphatases

**FIGURE 3:**
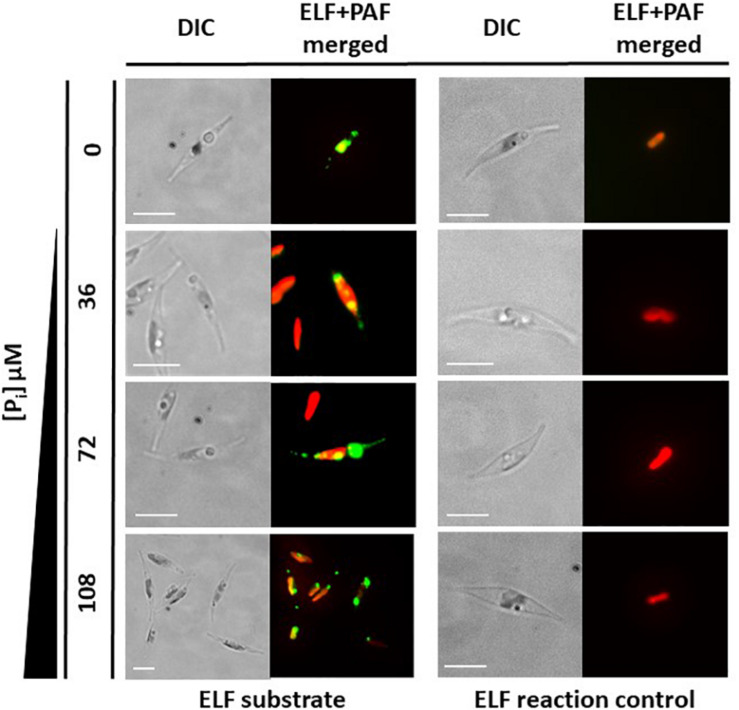
*In vivo* enzyme-labeled fluorescence (ELF) assays. Wild-type cells grown under different inorganic phosphate (P_i_) concentrations for 48 h showed fluorescence (green) emitted through the reaction of phosphatases with ELF substrate. Chloroplast autofluorescence is shown in red. The ELF reaction does not occur without the substrate (ELF reaction control). Scale bar: 10 μm.

A classical test to identify phosphatase activity is the use of ELF 97 phosphatase substrate. This substrate can be hydrolyzed by phosphatases, forming a yellow–green fluorescent precipitate that can be visualized (e.g., Dyhrman and Palenik, 1999). We tested *P. tricornutum* cultures with this substrate and observed that a fluorescent precipitate is formed by *P. tricornutum* cells, grown in P_i_-limiting f/2 medium and P_i_-supplemented medium (Figure 3). However, the precise cellular localization of the phosphatases could not be identified in these experiments.

Next, we expressed PtPhos3, PtPhos5, PtPhos6, PtPhos7, and PtPhos8, predicted to be membrane proteins, as eGFP fusions *in vivo* to study the localization of these proteins. For all the predicted candidates, *eGFP* was cloned at the 3′ end of the phosphatase genes so that eGFP would be located at the C-terminus when expressed as a fusion protein. For PtPhos6, we additionally designed an expression vector in which eGFP was fused at the N-terminus to avoid a putative masking of the ultimate C-terminal membrane domain. For these and all other localization experiments in this study, the expression of the candidate proteins was under the control of the nitrate reductase (*NR*) promoter to avoid any P_i_-dependent regulatory effects of the endogenous promoters. In addition, *P. tricornutum* clones obtained after transformation were assessed for gene integration *via* colony PCR.

**FIGURE 4:**
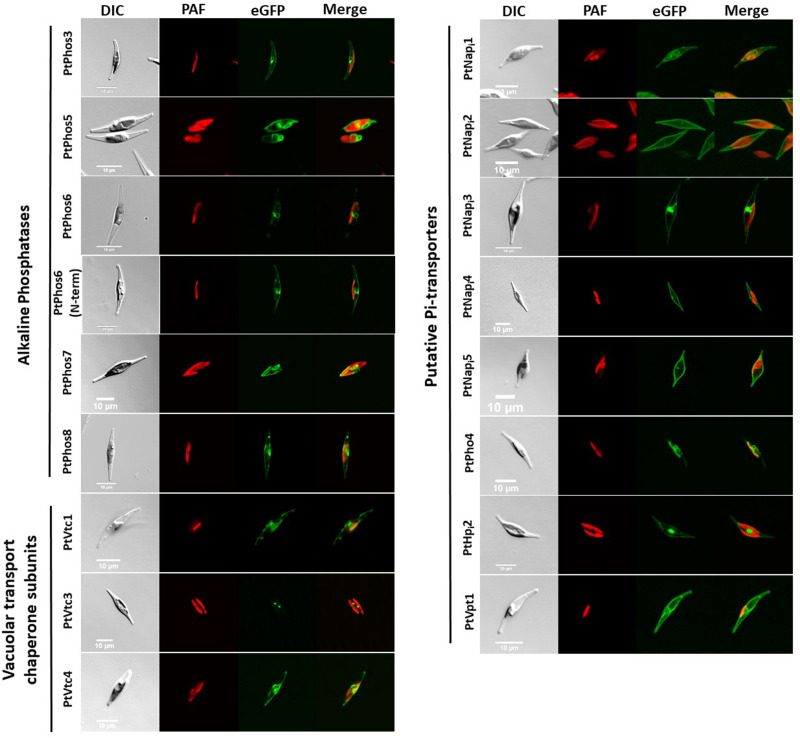
*In vivo* localization of eGFP fusion phosphatases, P_i_-transporters, and VTC subunits. DIC: transmission light; PAF: plastid autofluorescence; eGFP: eGFP fluorescence. Scale bar 10 μm. The expression of eGFP-fusion proteins was performed incubating cells in f/2 containing 0.9 nM NaNO_3_ instead of 1.5 nM NH_4_Cl for 24 h in sterile reaction tubes in the conditions described in section “Materials and Methods.” Localization studies were performed using confocal laser scanning microscopy using a Leica TCS SP2 with an HCXPL APO40/1.25-0.75 oil CS objective.

As shown in Figure 4, PtPhos5- and PtPhos7-eGFP fusion proteins were not integrated into the plasma membrane of the diatoms, but instead integrated into an internal cell membrane (henceforth referred to as the “endomembrane system”), which included the endoplasmic reticulum (ER) membrane, spanning the host ER, nuclear envelope, and outermost plastid-surrounding membrane (chloroplast ER, cER membrane) (Figure 4). PtPhos8-eGFP, which was upregulated under P_i_-depleted conditions, was integrated into the plasma membrane. Notably, PtPhos8-eGFP localization was not limited to the plasma membrane, as endomembrane localization was also detected under the conditions used (Figure 4). The same dual pattern was detected for PtPhos3-eGFP, which was not, or only slightly, transcriptionally regulated by P_i_ concentration. As mentioned above, PtPhos6 might be membrane-localized *via* a C-tail anchor. However, no difference was observed when expressing eGFP fusions in which the fluorescence marker was located at either the N- or C-terminus; for both versions, an endomembrane localization, most likely ER membrane, was detected (Figure 4).

### Phosphate Delivery Into the Cell and Intracellular Distribution

Dissolved inorganic phosphate, if needed, must be internalized and distributed to the different cellular compartments. *P. tricornutum* expresses several P_i_-transporters. Based on predicted domains, we studied a phosphate permease (PtPho4), two P_i_/H^+^ cotransporters (PtHp_i_1 and PtHp_i_2), and six Na/P_i_ symporters (PtNap_i_1–PtNap_i_6) (Table 1). Many of these putative transporters (PtPho4 and PtNap_i_2, 3, 4, 6) were significantly upregulated as indicated in published transcriptional datasets (Yang et al., 2014; Cruz de Carvalho et al., 2016; Alipanah et al., 2018). Several P_i_ transporters possess an SPX (SYG1/Pho81/XPR1) domain, important for the regulation of these proteins (Duan et al., 2008; Hürlimann et al., 2009; Secco et al., 2012a, b). As no SPX domain is encoded in the protein structure of the aforementioned phosphate transporters, we additionally screened the *P. tricornutum* genome for such a domain and identified an additional putative transporter (PtVpt1, Phatr2_19586).

The published transcriptional expression datasets for phosphate transporters are sometimes inconclusive. In addition, upregulation of a transcript can be interpreted either as upregulation from a basal expression level or as expression resulting from P_i_ limitation. To differentiate between these two possibilities, we investigated the expression of eGFP under the control of the promoter and terminator regions of *PtPho4* and *PtNap_i_2*. Both putative transporters have been reported to be upregulated under P_i_-limited conditions (Yang et al., 2014; Cruz de Carvalho et al., 2016; Alipanah et al., 2018). In parallel, *PtNap_i_4*, upregulated under P_i_ depletion (Yang et al., 2014; Cruz de Carvalho et al., 2016; Alipanah et al., 2018), and *PtHp_i_1* were also tested using the same strategy. As shown in Figure 2, the regulatory regions of both Na/P_i_ transporters induced a maximum expression level under conditions of P_i_ limitation; however, a basal level of eGFP protein expression was also detected with P_i_ supplementation. In contrast, *PtPho4* expression was strictly P_i_-dependent because the promoter/terminator regions induced eGFP expression only under conditions of phosphate limitation. Gene expression data for *PtHp_i_1* are not consistent according to the available RNA-seq data (Yang et al., 2014; Cruz de Carvalho et al., 2016; Alipanah et al., 2018). However, in our study, the promoter/terminator regions of *ptHp_i_1* induced eGFP expression under all P_i_ concentrations (Figure 2), with a possible lower level under P_i_-depleted conditions. The results for the phosphatase regulation experiments and the eGFP fluorescence confocal microscopy data are shown in Supplementary Figure S1.

Next, we analyzed the subcellular localization of eGFP fused with the P_i_ transporters PtNap_i_1–PtNap_i_5, as well as that of eGFP fused with PtHp_i_2 and PtPho4 (Figure 4). PtNap_i_2, PtNap_i_4, and PtNap_i_5 localized to the plasma membrane, whereas PtPho4 and PtNap_i_1 showed a potential localization at endomembrane reminiscent of the ER and nuclear envelopes. PtNap_i_3- and PtHP_i_1-eGFP signals were observed both in the plasma membrane and in a distinct, spot-like structure. Notably, we have not analyzed a further NaP_i_-type transporter (NaP_i_6), which is mentioned in Table 1.

### Vacuolar Transport Chaperone Complex and a Vacuolar Phosphate Transporter

**FIGURE 5:**
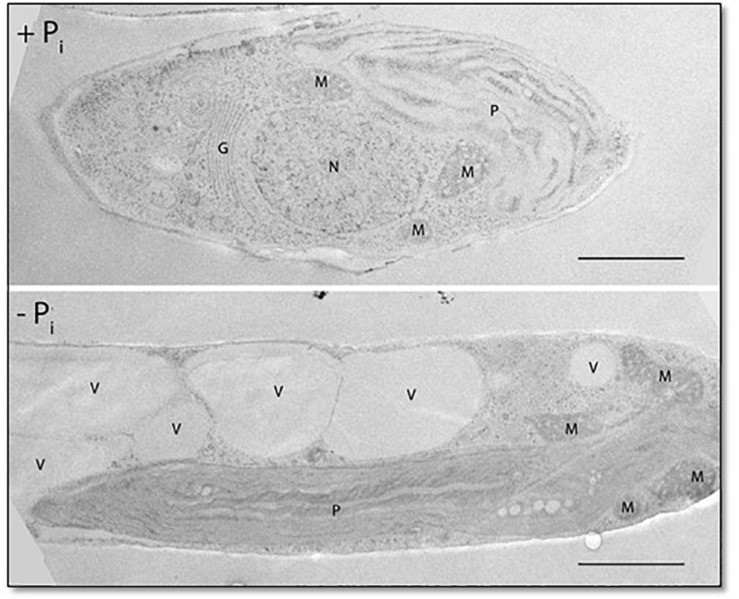
Fifty-nanometer ultrathin sections of *P. tricornutum* grown under P_i_-replete and deplete conditions for 48 h. P, plastid; M, mitochondrion; G, Golgi apparatus; N, nucleus; V, vacuole. Scale bar: 1 μm.

Cellular P_i_ stores might be important for phosphate homeostasis. Vacuoles can act as phosphate storage compartments, as observed for storage of polyphosphates (polyPs) in algae and yeast (Yang et al., 2017; Sforza et al., 2018). In *P. tricornutum*, vacuole morphology is dependent on the phosphate concentration in the culture medium, as observed in cultures growing under conditions of P_i_ depletion in which the volume of the vacuole is significantly increased in comparison with cultures grown in P_i_-containing media (Figure 5).

The vacuolar transport chaperone (VTC) complex, which consists of at least four subunits (Vtc1–Vtc4; Secco et al., 2012a, b), is an important protein complex involved in yeast polyphosphate synthesis. We have previously shown that the Vtc2 (referred to here as PtVtc2) subunit localizes to the vacuolar membrane, especially to sites at which two vacuolar membranes abut (Schreiber et al., 2017). Besides PtVtc2, *P. tricornutum* also encodes Vtc1, Vtc3, and Vtc4 homologs. All the subunits were only moderately transcriptionally regulated, if at all, following phosphate limitation (Table 1). We expressed PtVtc1, PtVtc3, and PtVtc4 as eGFP fusions, and no vacuolar localization was observed (Figure 4). However, for PtVtc3, the eGFP signal was found in defined foci, as previously reported for PtVtc2 localization (Schreiber et al., 2017). Therefore, we additionally analyzed the localization of PtVtc3 using immunogold labeling that showed no vacuolar localization (Supplementary Figure S4). Differently composed VTC complexes have been reported to exist, with one being shown to relocate from the ER/nuclear envelope to the vacuolar membrane with P_i_ deficiency (Yang et al., 2017). However, we did not observe this in the diatom, in which PtVtc1 and PtVtc4 were endomembrane-localized independently of phosphate availability in *in vivo* experiments (data not shown).

A prerequisite for a P_i_ storage function of the vacuole might be a phosphate transporter. We identified PtVpt1 *in silico* through its SPX domain. *PtVpt1*, a homolog of plant vacuolar P_i_ transporters (Liu et al., 2015; Liu T.-Y. et al., 2016; Alipanah et al., 2018), is upregulated under P_i_-depleted conditions (Alipanah et al., 2018). We expressed eGFP under the control of the promoter/terminator regions of *PtVpt1* and identified a strong upregulation under P_i_-limited conditions and a basal expression in the presence of P_i_ in the media (Figure 2 and Supplementary Figure S1). As expected, the PtVpt1-eGFP fusion protein showed a typical vacuolar localization pattern when expressed *in vivo* (Figure 4).

## Discussion

Phosphate is a key molecule for the synthesis of many essential biomolecules such as phospholipids and nucleic acids, whereas reversible phosphorylation can regulate the activity of numerous proteins. Several systems have evolved for the uptake of phosphate from the environment, including cooperation between two organisms, as occurs with mycorrhiza–plant symbioses (e.g., Wang et al., 2017). Diatoms can mobilize phosphate from the environment and deliver it to the different compartments of the cell. Although the cellular mechanisms involved in P_i_ mobilization and intracellular distribution in diatoms are not well understood, several studies have investigated whole-cell transcriptomic responses concerning phosphate deprivation (Dyhrman et al., 2012; Yang et al., 2014; Shih et al., 2015; Cruz de Carvalho et al., 2016; Zhang et al., 2016; Alipanah et al., 2018). Some of these responses might be general stress indicators, others specific for P_i_-limited conditions. For example, a comparative investigation indicated that deficiency of either phosphorus or nitrogen led to some similar responses with respect to central carbon and amino acid metabolism, whereas some P_i_ transporters are regulated only under P_i_ deprivation (Alipanah et al., 2018). This indicates that transcriptomic data are excellent tools to use for the reconstruction of cellular responses related to phosphate limitation, which is especially true for affected pathways with defined cellular localization. However, missing data on the localization of candidate proteins hinder more detailed analyses. For example, no detailed conclusions can be deduced about transporters if the membrane, in which they act, is unknown. Here, we aimed to close this gap of knowledge with respect to P_i_ mobilization and intracellular distribution of several transcriptional P_i_-regulated and non-regulated phosphatases and P_i_ transporters in the diatom *P. tricornutum*.

We first searched published data, including the genome sequence of *P. tricornutum* (Bowler et al., 2008; Rastogi et al., 2018), to identify putative essential players in phosphate assimilation. This led to the identification of candidates, which, according to several transcriptional studies (Yang et al., 2014; Cruz de Carvalho et al., 2016; Alipanah et al., 2018), are either phosphate regulated or not. For candidates with inconsistent expression data, we investigated the potential of their promoter/terminator regions to drive eGFP expression under P_i_-replete and -depleted conditions. Moreover, we evaluated the localization of candidate proteins within the cell, or as being secreted.

Taken together, our data showed three levels of activity important for phosphate homeostasis in the diatom: (i) extracellular and intracellular phosphate mobilization, (ii) phosphate uptake, and (iii) transport/export of P_i_ into/out of organelles.

### Extracellular Phosphate Mobilization

The P_i_ concentration of the environment can be increased by phosphatases, enzymes that catalyze the hydrolysis of a phosphoric acid monoester/diester, thereby providing P_i_ for further uses. We identified four enzymes acting most likely extracellularly (Figures 1–4): PtPhos1, 2, 3, and 8. Two of these, PtPhos1, a PhoA-like phosphatase, and PtPhos2, having a predicted phytase-like domain, are secreted enzymes (Figure 1), first identified by analyzing the medium for secreted proteins (Lin et al., 2013; Buhmann et al., 2016; Erdene-Ochir et al., 2019). Interestingly, both enzymes differed significantly in their molecular mass determined by Coomassie-stained SDS–PAGE gels and the use of prediction tools, respectively. The most likely explanation for this is related to their different isoelectric points (4.62 for PtPhos1 and 3.74 for PtPhos2). As further confirmation of this hypothesis, we expressed cDNA encoding *PtPhos1* tagged with a C-terminal FLAG, which again resulted in the detection of proteins presenting a higher molecular mass (100/110 kDa) in Coomassie-stained gels when compared with the calculated values. Moreover, posttranslational modifications of the proteins might also have contributed to the mass shift. PtPhos1 was previously shown to be secreted into the medium (PtAPase, Lin et al., 2013) and modulated by phosphate concentration (Lin et al., 2017). Analysis of the promoter and terminator regions of the *PtPhos1* gene showed that these regions induced a strong upregulation of eGFP expression under P_i_-depleted conditions and a basal expression pattern in P_i_-containing medium. In principle, a basal expression level in media having an initial phosphate concentration of 36 μM could have been induced by an early starvation response, caused by P_i_ consumption of the cells (for cell concentration at sampling point, see Supplementary File S1 and Supplementary Table S3). For this reason, we have also tested the expression at higher P_i_ concentration. And indeed, the expression of *PtPhos1* and *PtPhos6* displayed a pattern that might indicate this assumption. However, the other genes showed either a constant basal level or a complete repression of expression (Figure 2). In case the weak signals obtained in the cultivation of *PtPhos1* and *PtPhos6* cell lines in 36 μM P_i_ might be caused by a weak P_i_-stress response that did not occur at higher P_i_-concentrations, expression of *PtPhos1* and *PtPhos6* might be controlled not only by the external P_i_ concentration alone, but also by other factors (e.g., intracellular P_i_ pools, PtPSR transcription factor activity; Sharma et al., 2019), which makes them differently sensitive to the experimental setting. Protein expression and delivery to the final destination can be controlled not only through transcriptional regulation, but also during translation or by posttranslational regulation. By analyzing the amino acid sequence of PtPhos1 *in silico*, we identified several predicted phosphorylation sites in the N-terminal region of the mature protein. As these putative targets for posttranslational modification were not identified in PtPhos2 (Supplementary Figure S2) and secretion of the latter involves transcriptional regulation, we used phospho-mimicry to investigate whether reversible phosphorylation of PtPhos1 might control its secretion. We did not detect any differences in the secretion of the protein under different phosphate concentrations in the medium. However, other factors and/or mechanisms might be involved in regulating PtPhos1 secretion, which requires further investigation.

PtPhos2, first identified in a screen for secreted proteins by Buhmann et al. (2016), was either found to be P_i_-regulated or not identified as a member of a group of P_i_-regulated genes (Yang et al., 2014; Cruz de Carvalho et al., 2016; Alipanah et al., 2018). To investigate whether PtPhos2 expression is indeed P_i_-dependent, we used the upstream promoter and downstream terminator regions of its gene to express eGFP under differing phosphate availability (Figure 2). We detected an eGFP signal only under phosphate-limited conditions, indicating that the regulatory regions of *PtPhos2* might be useful also for regulated gene expression in synthetic approaches. This was also recently proposed by another group; however, P_i_ regulation was not observed, most likely because of the small size of the analyzed upstream region (499 bp) (Erdene-Ochir et al., 2019).

The immediate environment of a cell in a fluid medium, such as that of a diatom in the ocean, is not uniform. Instead, the cell is surrounded by a layer that has a higher viscosity than the surrounding fluid. Nutrients in this layer can be taken up by the cell, thereby creating a nutrient-depleted region (Pasciak and Gavis, 1974). Fine-scale turbulence can distort this layer, such that components of the surrounding medium can diffuse more easily to the cell surface (Karp-Boss et al., 1996), and diatoms can benefit from this boundary-layer distortion by increasing phosphate uptake from the medium (Peters et al., 2006; Dell’Aquila et al., 2017). However, microturbulence can also result in molecules drifting out of the layer, including secreted phosphatases. Therefore, it would be advantageous to express a second set of phosphatases that are anchored to the cell surface to provide additional activity within the boundary layer. To identify such enzymes, we first searched for the phosphatase activity of potential plasma membrane-localized enzymes using an ELF assay, a fluorescence-based method commonly used to assay for phosphatase activity (González-Gil et al., 1998). Using this assay, we detected phosphatase activity under both P_i_-limited and P_i_-replete conditions. However, the signals were not specific in terms of their cellular localization, and consequently, we could not provide evidence for cell surface–localized phosphatase activity by this assay (Figure 3). Nevertheless, we identified two putative phosphatases (PtPhos3, PtPhos8) that localized to the plasma membrane, one of which (PtPhos8) may possess an extracellular catalytic domain based on topology predictions. This protein might be responsible for the cell surface phosphatase activity reported in Flynn et al. (1986). Nevertheless, the presence of secreted and plasma membrane-anchored phosphatases, whether P_i_ regulated or not, should enable *P. tricornutum* to scavenge P_i_ under different environmental conditions. This may be especially important for *P. tricornutum*, for which phosphonates are not the ideal source of P_i_ uptake into the cell (Whitney and Lomas, 2019).

### Intracellular Phosphate Mobilization

Under P_i_-limited conditions, phosphate can be mobilized from intracellular resources, either from P_i_ stores or by exchange of P for non-P biomolecules. As shown for other organisms, the vacuole of the diatom can act as a P_i_ store. We analyzed the morphology of the vacuole and noticed that the volume of the vacuole increased significantly under P_i_-limited conditions (Figure 5). Notably, vacuole enlargement is not restricted to conditions of P_i_ limitation, as previously shown in a *P. tricornutum NR* knockout strain propagated in NO_3_^–^-containing medium (McCarthy et al., 2017).

The VTC complex is involved in vacuolar phosphate metabolism in several unicellular eukaryotes (e.g., Yang et al., 2017), in addition to its other functions (e.g., Uttenweiler et al., 2007). We have previously shown that one VTC subunit, PtVtc2, preferentially accumulates in regions in which two vacuolar membranes are in close proximity (Schreiber et al., 2017). We expected to find the same cellular localization for the identified homologs of PtVtc1, PtVtc3, and PtVtc4. In contrast to that observed for PtVtc2, the other PtVtc-eGFP fusion proteins exhibited an endomembrane localization (Figure 4). Interestingly, a VTC complex is known to relocate from the nuclear envelope/ER to the vacuole under conditions of phosphate limitation (Yang et al., 2017). However, PtVtc1 and PtVtc4 localization did not change with varying P_i_ availability. This suggests that differently composed VTC complexes may have different functions in *P. tricornutum*. If one of the functions of the *P. tricornutum* vacuole is to store P_i_, a phosphate transporter would be expected to be present in the vacuolar membrane. This was indeed the case, as we found that PtVpt1, a homolog of plant vacuolar transporters, was localized in the vacuolar membrane and found to be transcriptionally regulated according to P_i_ availability (Figures 2–4). Nonetheless, we could not confirm a P_i_-storage function for the vacuole of the diatom.

We identified PtPhos5, PtPhos6, and PtPhos7 as phosphatases, which, when expressed as eGFP fusion proteins, localized to the endomembrane system (Figure 4). However, their catalytic domain might be exposed either to the lumen of the ER or cytoplasm.

Interestingly, as shown for *Thalassiosira pseudonana* (Martin et al., 2011), diatoms substitute phospholipids with non-P lipids when grown under P_i_-limited conditions. Similarly, a significant reduction in phospholipids and an increase in betaine lipid content have been reported for *P. tricornutum* (Cañavate et al., 2017a, b; Huang et al., 2019). This suggests that endomembrane-localized phosphatases might function in the degradation of phospholipids and especially in hydrolyzing P_i_ from lipase-catalyzed phospholipid degradation products under P_i_-limited conditions. PtPhos6, which is strongly induced under P_i_-limited conditions (Figure 2), is an ideal candidate to perform this potential P_i_ “recycling” activity.

### Phosphate Uptake

Six putative transporters (PtHp_i_2, PtNap_i_2, PtNap_i_3, PtNap_i_4, PtNap_i_5, and PtNap_i_6) were identified as being involved in phosphate uptake from the medium, and PtNap_i_2–PtNap_i_5 localized to the plasma membrane when expressed as eGFP fusion proteins (Figure 4). According to published transcriptional data, the expression of two of these analyzed genes, *PtNap_i_2* and *PtNap_i_4*, is either significantly (*PtNap_i_2*) or moderately (*PtNap_i_4*) regulated by P_i_ availability. To identify whether both putative P_i_ transporters are expressed only under P_i_-limited conditions or are upregulated under these same conditions, we assessed eGFP expression when driven by their respective promoter/terminator regions (Figure 2 and Supplementary Figure S1). The regulatory regions of both genes induced strong upregulation of eGFP expression under P_i_-limited conditions and a basal level of expression in P_i_-replete media. Thus, for phosphate homeostasis, several transporters are targeted to the plasma membrane in the diatom to increase phosphate uptake under P_i_-limited conditions. However, as several transporters such as PtNap_i_2 and PtNap_i_4 (Figure 2) are also expressed under P_i_-replete conditions, we cannot exclude that these proteins undergo posttranslational modifications for a finer regulation of P_i_ uptake. Notably, the upstream region of *PtNap_i_4* (Phatr47667) was recently proposed as a novel strong promoter for the expression of lipogenesis-related genes for industrial applications (Zou et al., 2019). In this study, we could not determine the subcellular localization of PtHp_i_1. However, using both the upstream and downstream regions to drive eGFP reporter expression, we observed a P_i_-constitutive expression with a possible slight downregulation of the protein under P_i_-depleted conditions (Figure 2). These results are consistent with the transcriptional pattern observed by Alipanah et al. (2018). However, transcriptomic data concerning this gene under P_i_ starvation are inconsistent according to other datasets (Yang et al., 2014; Cruz de Carvalho et al., 2016) in which a downregulation was reported, although this suggested that PtHp_i_1 may function as a low-affinity P_i_ transporter.

### Phosphate Distribution

As mentioned above, PtVpt1 possesses an N-terminal SPX domain that is likely to be exposed to the cytosol. These domains are known to be involved in P_i_ homeostasis *via* interaction with inositol polyphosphate signaling molecules (Wild et al., 2016). We could not assign a complete functional description to this transporter based solely on a determined subcellular localization; however, we hypothesize that this protein has a role in P_i_ influx/efflux in the vacuole, and the function of this protein might be both directly and indirectly controlled by the intracellular/extracellular P_i_ levels *via* the SPX domain.

We identified transporters in the endomembrane system, two of which (PtNap_i_1 and PtPho4) were likely localized to the ER/nuclear envelope (Figure 4). Several biomolecules with important phosphate groups have to be exchanged between the plastid and the host, such as (deoxy-) purine nucleotides (Ast et al., 2009; Chu et al., 2017) and triosephosphates. We recently investigated triosephosphate transporters and showed that at least one is located in each of the different plastid-enveloping membranes (except for the second innermost membrane, homologous to the outer envelope membrane of chloroplasts) (Moog et al., 2015). Triosephosphate transporters mediate counter-exchange of P_i_ with triosephosphates (phosphorylated sugars), and there is no net import of P_i_ into plastids. However, we identified two endomembrane-localized phosphate transporter candidates, PtPho4 and PtNap_i_1, one of which (PtPho4) was highly upregulated following phosphate limitation and repressed under P_i_-replete conditions (Figure 2 and Supplementary Figure S1). These two putative transporters might be involved in P_i_ exchange between the host and the plastid in diatoms. The localization of PtPho4 was surprising because a close homolog in *Saccharomyces cerevisiae*, Pho89, is a repressible, high-affinity phosphate transporter present at the plasma membrane (Secco et al., 2012a, b). Although our data do not support that PtPho4 is a plasma membrane-localized transporter, we cannot exclude that this protein undergoes phosphate-regulated posttranslational modifications, which might eliminate it from the plasma membrane under P_i_-replete conditions. However, in such a scenario, PtPho4 would be expected to be targeted to a degradation compartment, such as the vacuole, and not to the ER. Thus, the ER localization of PtPho4 seems to be correct.

## Conclusion

Diatoms regulate their phosphate requirements at different cellular levels. We analyzed transcriptionally P_i_-regulated and -non-regulated factors, expected to be involved in phosphate mobilization, import, and intracellular distribution, with respect to their expression and localization. Our results indicated that P_i_-upregulated factors act in the extracellular space, at the plasma membrane, vacuole, and endomembrane system, which includes the cER, precisely the cellular positions essential for the regulation of phosphate homeostasis in diatoms. Therefore, the already published “omics” data, combined with our *in vivo* localization and expression studies, provide comprehensive insights into the strategies adopted by diatoms in environments with fluctuating P_i_ availability. We also identified strongly P_i_-dependent promoter/terminator modules that can drive the expression of transgenes in the *P. tricornutum* model organism. The novelty of these expression modules lies in their ability to repress gene expression, making them suitable for certain experimental approaches that require a particularly fine level of transcriptional regulation.

## Data Availability Statement

The mass spectrometric proteomic data were deposited to the ProteomeXchange Consortium via the PRIDE partner repository with the dataset identifiers PXD016456 and 10.6019/PXD016456.

## Author Contributions

UM conceived this study together with GD and SZ. GD, FH, VS-G, and SR did the experimental work. TH performed the electron microscopy analysis. JK did the mass spectrometry analysis. The manuscript was written by UM and GD. All authors read and approved the manuscript.

## Conflict of Interest

The authors declare that the research was conducted in the absence of any commercial or financial relationships that could be construed as a potential conflict of interest.

## Footnotes

1 https://genome.jgi.doe.gov/portal/Phatr2/Phatr2.download.html

2 http://genome.jgi-psf.org/Phatr2/Phatr2.home.html

3 https://protists.ensembl.org/Phaeodactylum_tricornutum

4 http://www.cbs.dtu.dk/services/SignalP-3.0

5 http://www.cbs.dtu.dk/services/SignalP-4.1/

6 http://topcons.cbr.su.se

7 http://phobius.sbc.su.se/

8 http://www.cbs.dtu.dk/services/TMHMM

9 http://www.ch.embnet.org/software/TMPREDform.html

10 http://www.ncbi.nlm.nih.gov/Structure/cdd/wrpsb.cgi

11 https://web.expasy.org/peptide_mass/

12 http://www.dabi.temple.edu/disphos/

13 https://mycocosm.jgi.doe.gov/Phatr2/Phatr2.home.html
