# Supplementary material for: Mobilization and Cellular Distribution of Phosphate in the Diatom Phaeodactylum tricornutum
Source: Front Plant Sci. 2020 Jun 3;11:579. doi: 10.3389/fpls.2020.00579 (PMC7283521; doi:10.3389/fpls.2020.00579)
Supplement: Supplementary file 1 [file Data_Sheet_1.pdf]

Supplementary File 1

Mobilization and cellular distribution of phosphate in the diatom *Phaeodactylum tricornutum*

Gianluca Dell'Aquila [1], Stefan Zauner[1], Thomas Heimerl[2], Jörg Kahnt[3], Vera Samel-Gondesen[1], Simon Runge[1], Franziska Hempel[2], Uwe G. Maier[1,2,*]

* **Correspondence:** Prof Dr. Uwe G Maier: maier@biologie.uni-marburg.de

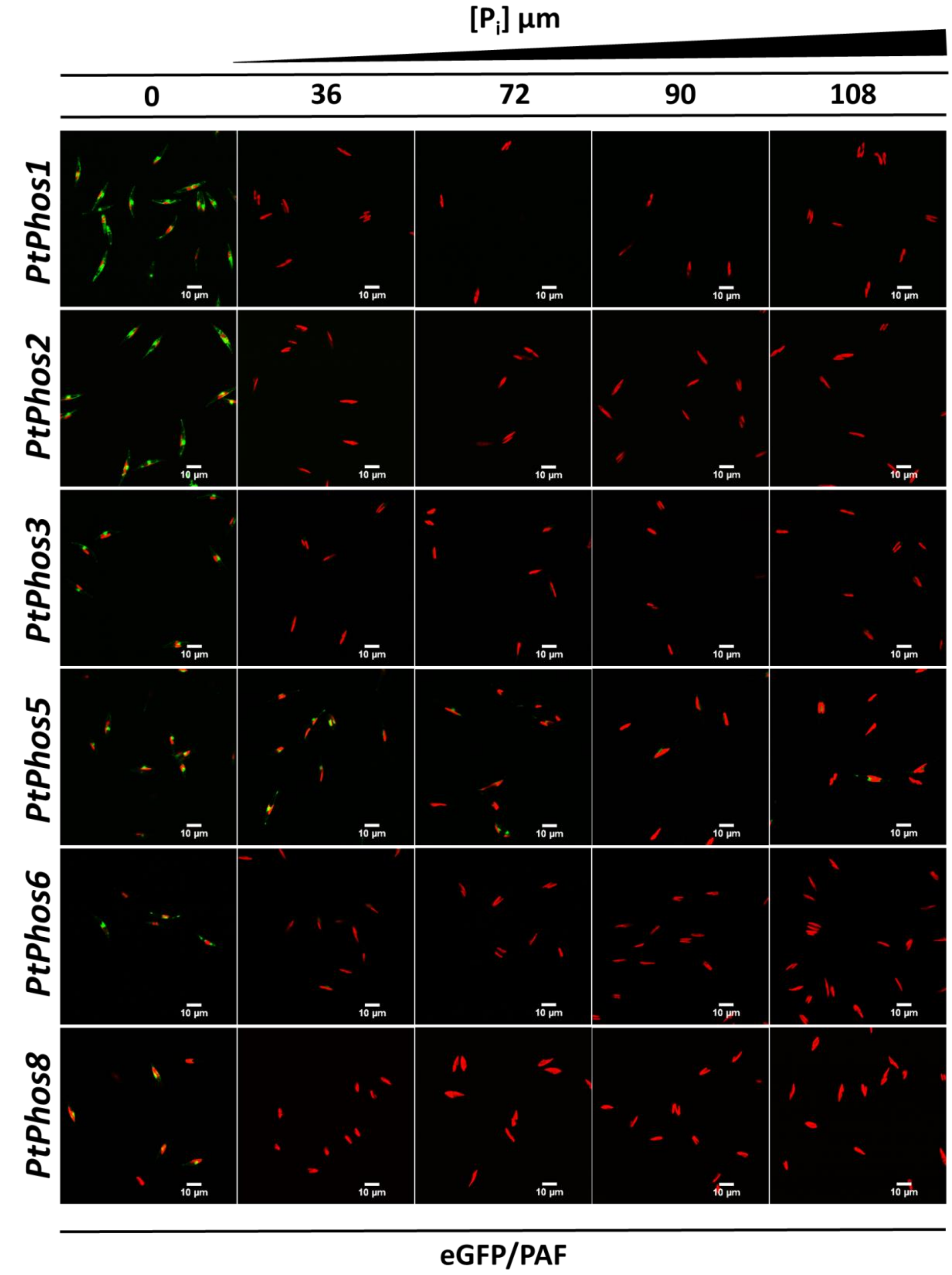

[Pi] µm
0
36
72
90
108
PtPhos1
PtPhos2
PtPhos3
PtPhos5
PtPhos6
PtPhos8
10 µm
eGFP/PAF

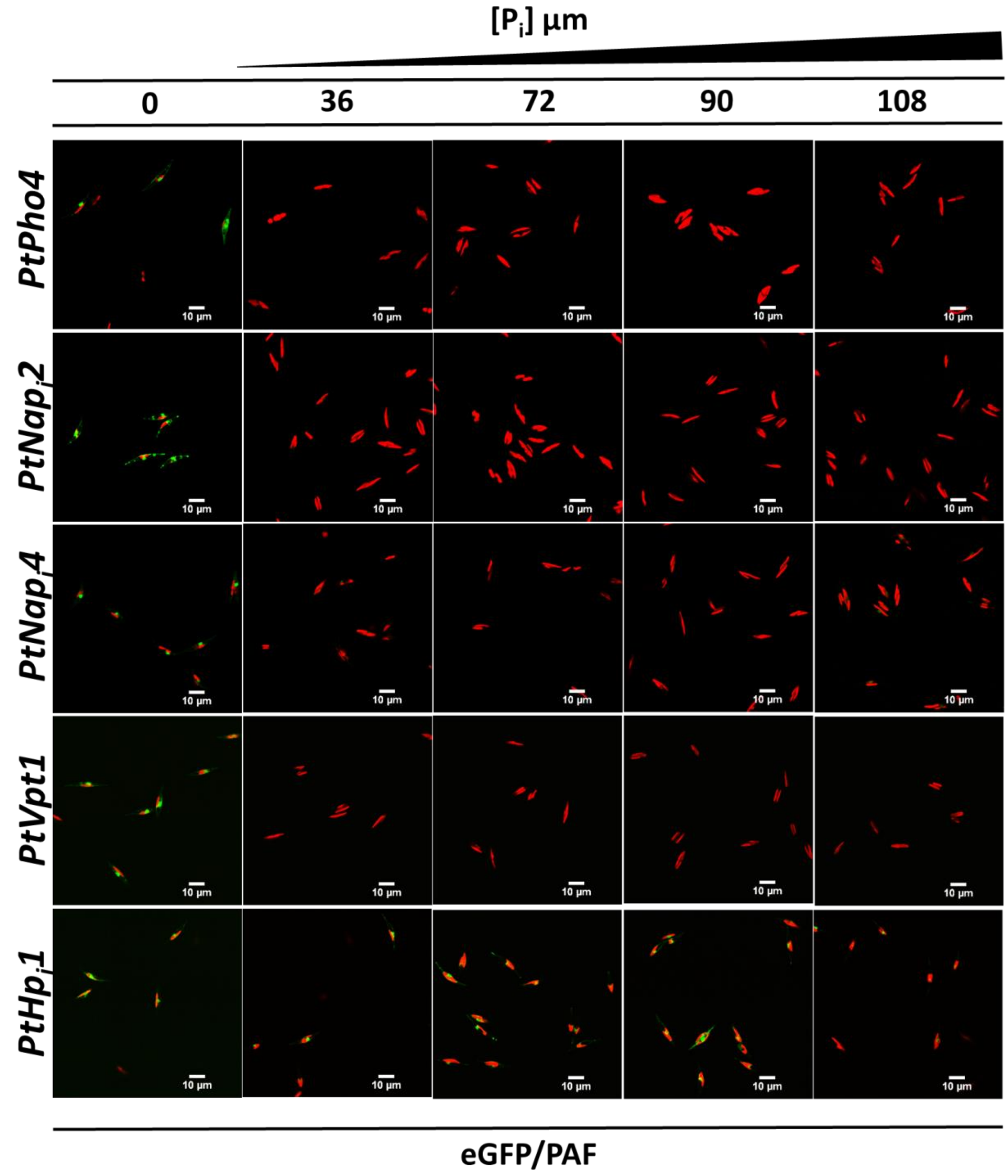

Supplementary figure S1: confocal scanner lase microscopy *in vivo* eGFP expression of different promoter/terminator cassette strains (denoted in the left side), incubated under different P_i concentrations for 48h. Figures are shown as merged pictures with eGFP (green) and autofluorescence (red). For each cell line, pictures were acquired using the same setting.

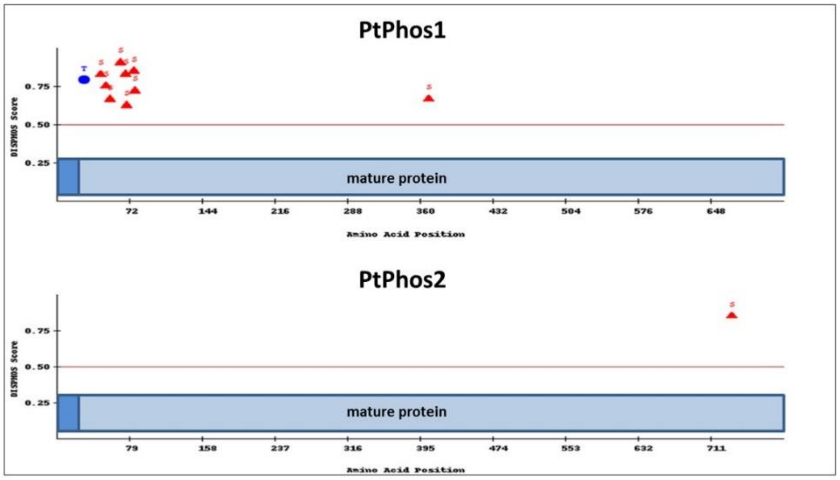

Supplementary figure S2: putative phosphorylation sites prediction using DISPHOS2 (http://www.dabi.temple.edu/disphos/). Predicted phosphorylation sites are shown with red triangle (serine) or blue circle (threonine).

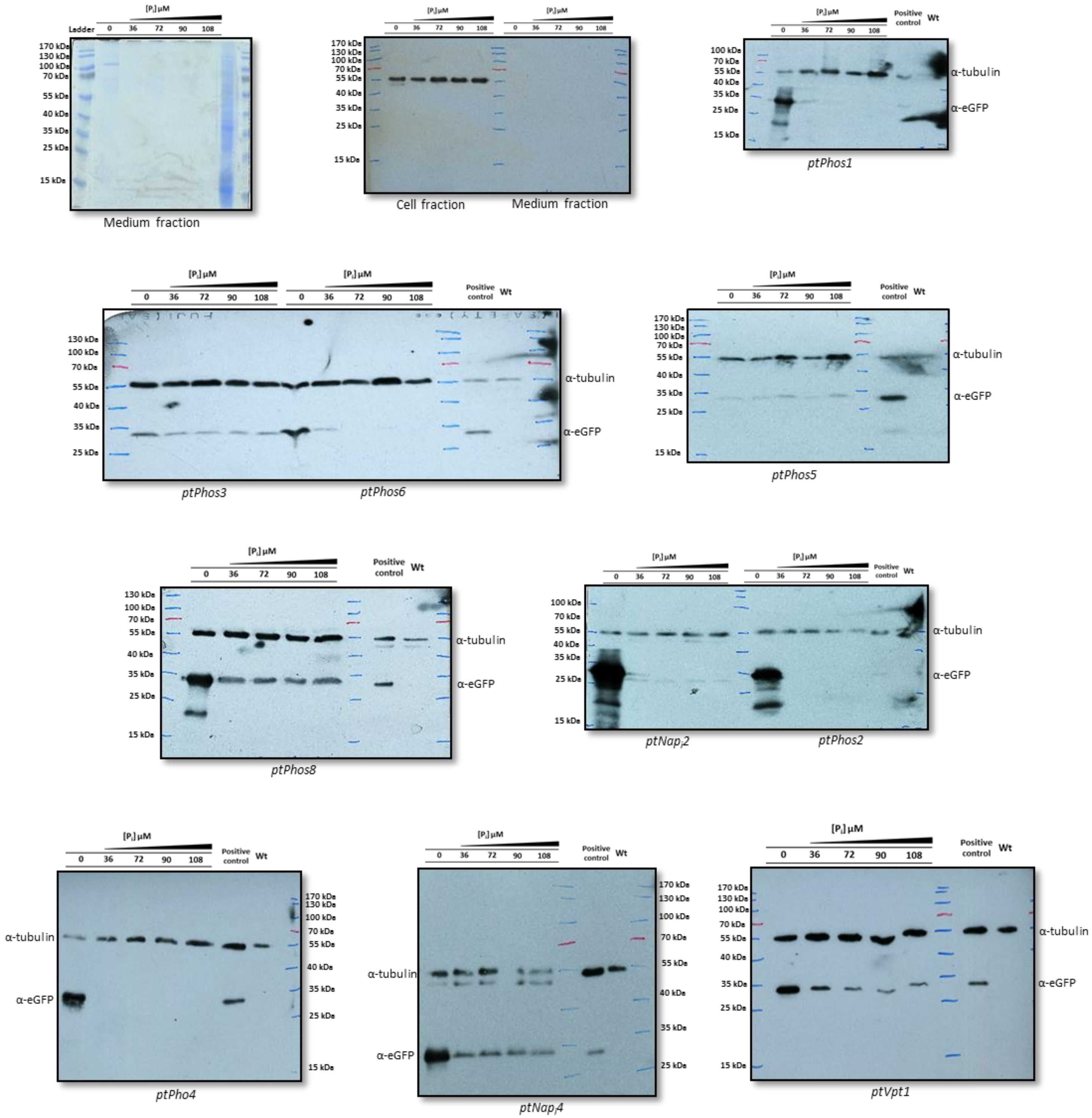

Supplementary figure S3: scans of all the blots and gels shown in this work. After protein transfer and blocking, membranes were cut at 40 kDa level and incubated separately with α-tubulin (upper part) and α-eGFP (lower part), as described in the material and methods section.

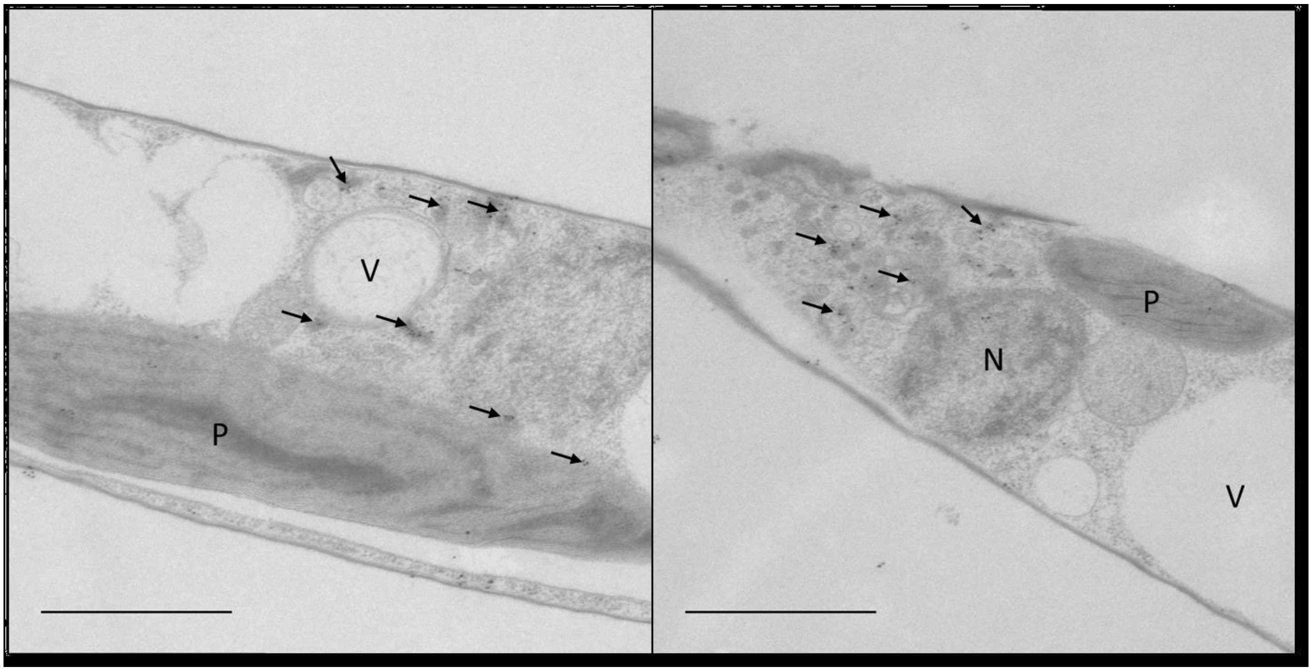

Supplementary figure S4: 50 nm ultrathin section showing PtVtc3-eGFP (black arrows). P: plastid; N: nucleus; V: vacuole. Scale bar 1 µm.

Running name	Forward	Reverse
PtPhos3	GAATTCATGGAGAGTCAGGGGAAGGAATGTCC	TCTAGAAAAGCTGTGAACTTGTGATGATAAAATTGTTCCCG
PtPhos5	GAATTCATGGCGGGTCCATCGAAACGGCCTTG	TCTAGACTTTTTGTTCTGCTCATTCC
PtPhos6	GAATTCATGAACGATGACCGGATAAGTGACCG	TCTAGACATCAATAATTTGGTGGACGGAACCG
PtPhos6-Nterm GFP	CAGGTCTAGAATGAACGATGACCGGATAAG	CTAGTCTTAAAGTAAATTGAAGCTTTTACATCAATAATTTGGTGGAC
PtPhos7	GTCACCACTTGTGCGAACGGAATTCATGAGCCGTGCGCCAACG	CCTCGCCCTTGCTCACCATTCTAGAGTCGATTTTGAGTTGACGCTTGTTGG
PtPhos8	GTCACCACTTGTGCGAACGGAATTCATGACGCAAATCTTGATCTTG	CCTCGCCCTTGCTCACCATTCTAGAAGCTAGACCATCTACGGTC
PtPho4	GAATTCATGAGCGTTGACATGAG	TCTAGAGGCAGAGGGGGAAAAAG
PtHpi1	GTCACCACTTGTGCGAACGGAATTCATGGAACCATCGGACAAATTG	CCTCGCCCTTGCTCACCATTCTAGAAACAGTATCTGCGAGAAC
PtNapi1	GAATTCATGGTAAGTTGCCGTCTTCATATTGAGTGTG	TCTAGACGCCTCGACTTCGTTGTCGTC
PtNapi2	GAATTCATGTCTGCAGAAAATCCTGAGG	GGATCCAGCTGCAACCTCATCTGAGTCCG
PtNapi3	GTCACCACTTGTGCGAACGGAATTCATGGAGATCAACAACGCC	CCTCGCCCTTGCTCACCATTCTAGATGTTATTCTCAAACAGTATCTGC
PtNapi4	GAATTCATGTCCAACGCACAATTCTTCTGTG	TCTAGAGGCATCAACCTCAGCATCCGAAGTATC
PtNapi5	GAATTCATGAATGTCATCCACGAGACCG	TCTAGAGGATTTTCAACGGTAT
PtVpt1	GAATTCATGGTGAACTTTGGCAATAAG	TCTAGAGTCATCATTGTCTAAAGGCTC
PtVtc1	GAATTCATGAGCCATTCTGAAACGACGCCG	TCTAGACGCTTCAATCATCGAATAC
PtVtc3	GAATTCATGCCGCCGCAACAGTCCCGAG	TCTAGATACAGTCGCCGCAGCGCATTCG
PtVtc4	GAGCTCATGAAGTACGGTGAACACC	TCTAGACATTAGGTCCATTTTCGAAATGG

Supplementary table S1: primers used to amplify the genes for localization studies. Restriction sites sequences are underlined and sequences marked in yellow indicate the overlap generated to use the Gibson assembly reaction to generate the constructs.

Gel slice	Description	Coverage [%]	# Peptides	# PSMs	# Unique Peptides
~100 kDA	Predicted protein OS=Phaeodactylum tricornutum (strain CCAP 1055/1) OX=556484 GN=PHATRDRAFT_49678 PE=4 SV=1	24	15	50	15
~130 kDA	Predicted protein OS=Phaeodactylum tricornutum (strain CCAP 1055/1) OX=556484 GN=PHATRDRAFT_47612 PE=4 SV=1	12	7	22	7

Supplementary table S2: summary of the Mass Spectrometry analyses performed on the bands showed in figure 1. These results are relative to experiment ran using the Orbitrap Velos Pro mass spectrometer (Thermo Fisher Scientific) and analyzed using Proteome Discoverer 2.2 (Thermo Fisher Scientific).

P_i concentration	Cell concentration										
	PtPhos1	*PtPhos2*	*PtPhos3*	*PtPhos5*	*PtPhos6*	*PtPhos8*	*PtPho4*	*PtHp$_i$1*	*PtNaP$_i$2*	*PtNaP$_i$4*	*PtVpt1*
**- P_i**	3,7	2,4	3,0	3,1	3,7	3,4	3,2	3	3,6	3,1	3
**36 µM**	3,4	2,8	3,0	3,8	3,8	3,8	3,3	3,1	3,3	3,4	3,1
**72 µM**	3,5	2,8	2,5	3,0	3,3	3,7	3,2	3	3,4	3,3	3,3
**90 µM**	3,5	2,7	2,7	3,8	3,4	3,6	3,1	2,8	3,8	3,3	3
**108 µM**	3,3	2,6	2,5	3,4	3,3	3,4	3,2	3,2	3,6	3	3,3

Supplementary table S3: cell concentrations of strains used in the expression studies two days after inoculation in media having different P_i-concentrations (first column). 2×10^6 cells/ml cells were initially inoculated. The values are expressed as multiple of 10^6 cells/ml.
