# Supplementary material for: Mobilization and Cellular Distribution of Phosphate in the Diatom Phaeodactylum tricornutum
Source: Front Plant Sci. 2020 Jun 3;11:579. doi: 10.3389/fpls.2020.00579 (PMC7283521; doi:10.3389/fpls.2020.00579)
Supplement: Supplementary file 2 [file Data_Sheet_2.pdf]

Supplementary file 2: nucleotide sequences and length of the upstream and downstream regions used to design eGFP expression cassettes, obtained by Sanger sequencing of the fragments amplified from *P. tricornutum* (strain UTEX646). Untranslated regions (UTR) are underlined in blue.

>*PtPhos1* upstream region, 949 bp
CCGGTGTGTAAATATACGATACCCGGAGCCGCGTTGGAGATTCATTCACTGTCAACGCTC
GGCCACGGCAATTTGCCCTAATATCGTAACACATAAAATGGCGACGTTTCCCTGCACGAC
CATAATTTCGTTCACGGCAGGGATTTTGGCGTCGAGATTCGTTCATGGGACCAAAACTAG
CAGGAGGATTGGTTTTTCAGCATAATAGATTCGTGTCCCCGTTTCGATTTTTCTCTGCGGC
CGTCCCCACCGGACTCGAAAAAATTTCAAAGTTAGAGCATTTGGTGAAATCGTACGCCAA
GTTTCCCGCTAGAAATAATGTGGACCGAGGGTTAAGTGTCGCGCATGTGCGAAGAATGCG
CTGGGAGCTGACTTCCCGGCCGACTGTAATTAGAGAAGAACATAGATTAAAAAAAGACT
TGTCAATTCGGAAGACACACAAGTAGACACAGAATACTAAACAAGAAGAGAGAAGCATG
ACTCATCTTTTTAAGCATCATAATAGATGATTTGCAGGGCGTTTGTATGAGTCAGCCAAGT
GCAAATATGTTTTACGTGTCAACGAATCTTAAAGATCCATGACAAAAACGCCATACACCA
GAACCATGAAATGGACGCTTCGTTAATGTAAAGTGTCGAATCTTCAGTTCACATTTTTCAA
GATTCGCTAGATTCGACAGTGATAAATCATCTTTGTCAAAACAATCTTCATGGTTGTTGAA
CGATATACGGTCTATACGTCCTAAATCAGAGAACCGTTCACAAATTACAATTAGATTCTTT
AGAGAAACCATGACCAGGCTCACGTGTACTTTCTTACAATTGATGGGATCGGATGCAAGA
CCGAGGAAAAGATCTCATCTCTTGGGCGCACTGATAAAGCTCATAATTTTGTGAGTTGCC
ACCAAGGATTTTTCTATCGGCATCCTAACATTATCGGACCTGCAAA

>*PtPhos1* downstream region, 523 bp
TCGGATTCTTGCAAGCTTGAGCACAGGAATTTGAGACTTATATATGTTGCGTCCGAAATTT
ACAGTTAGGCTTTCGTCCGATTTTGCTATGTAGGCGGAAATAGCATAGAATCATGTTACA
CAGTTAAGGCACTCATAGTTGTACATAGTGTGGTGCATTTTAGAGCTGTTTGGAACAAGC
AAACATTTTGAGTGCAGATTCCCCCACCCATGGAATGTGAGCGTTGCTCGCGCAGAATGT
CGATTTTACAGTTAGTTGGAACGGTGCGCCCGCTGGAGCCGACTTTTGATTGGAAGTACT
GTTCTGAACTGGTACACGTCGAGTCATACCCTGCCCTTCTTACATCTTATTGAGAGACGAT
GGCACTTTGCGAACAAAGTGGTTGTGATGTTCTAACAGTAAATATAAAAGGCTGTAGGTA
GTTTACCTAGCTCTACACGCAAACGGAGGACCGCATCAATTCCGTCGGAGGCCGTTACCG
TGTTCGCTTAGGCTACCGGACCGGCCCGTCTTTAGGCACTC

>*PtPhos2* upstream region, 1042 bp
GACCTGCAATACACCTAAGCGGGCCTAGGGACTGTAAAACTCAACTCGCTTACGACGCTC
CCCACGGGTTAGACAGCGGATACGAGGACCTATGCTAACGCAATATTTGGATCAATATCT
TTCTAAAACAATGAGAAGAAGATTGTTCTACTACTAGTCCATCGGGCTTGAGAGGTCCAG
TTCACTGGACTACCGTTACCATGTTCCAGATAATCTTACTCACATATATGAGTCTTGTTCT
TTTAAGTATAAATCCCTAAAAAACTATAGAAATCCTAGAAGCAGTCAGTAAATGTAAATC
CATCACTAATGTAACTCTAAGTTCCCTCTACTAGTCAATTGTACAACATAAATTCTTCACT
GATTGTGACTTTGAATTTTAGGACACACTCTCTAGAGAGGATTGATAGTGAAAACCTTAT
TCATTGTCAGAGCTTAAGCCGGTCTGGTCTATCTTTCCACTGTCAAACAGCTCTTGATTGT
CGCCCGCGCGAAAATAGTAGCACTAACTGTAACTTCAAAATACAAAATGTTCTCTGTTAC
CATACAGTGAATGTAACTTTCGAATTGACAGTATTAGTAGTCGTATTGACAGTGAGGCAC
GCCCCTCAATGTGCGAGGTGGAAAATATACCAGCATGACAATGAATCTTGGAGATTCTTT
TGCTGTCATCAAGATTCACCGCCAAATCTTCAGGAACCTATCACGTCCACAGGCGATGTT
AATTCTTGAGTCGTCAAAACAAAGTCCTGTCCTACCTGTAGAAGTTGACAGCGAGCAATT
GTATGCAAACTTCTGACTTTGTTATAATAACATTAAAGGTAATTAAGTATCTTCAATTAGG
CATTTTGTCACTGTCAGTCCGTTCCGACAATATAGGTAGATTTGGAATGAATCTTTTCTAT

GCTGCTGCGAATCTTGTACACCTTTGAGGCCGTAGATTCTGTCCGACGAAGCGATAATTA
TTGCAAAATACATGGACTCATTATTTTGATTCGATTTCTTTTTGGTATCCGACTCGAAAAG
ATCCATCACGGCGAGC

>*PtPhos2* downstream region, 500 bp
GTATAGAAGGTCACGTAGCTAGAGAAAGAAAGAGTTAATCTCAAAAAGAGTCAAGTCCA
CGAACTTCAAATAAACCATAAGTCTTCTTTCGTTTACCCATTGGCCTTCATCCTTCCAATC
AACACTCGATTCTGAACAGAAAATTGGACCGGGAATGATTCCTGCGATACTGTAAGCGAT
TTCCCTACCTGATTCCTCGATGCCATTAAATCTTCGGCAGCTGCGCGGCGCATTGCGTTCA
GTGCTCTAATCGCCCTATGCTGTCCACCAGATGAAGCTCCGGTTGAAAGAACCGAAACAC
GCCTAATATCGTCACGAAATTTTTCTAATGACTTTTGGTTGGCATCGCTGCGAAACATGAA
ATCAGCACGACGGGTCAAACACGCCAGAATCTGGTTTGACCTGAAGCAGCAAGTAGATA
TACAGAACGAAGTCTGTGCTCGAACAGCAATTTCTACAGCAGCTTCTGCAAGGCCTCCGC
AGCAATGGAGACCTCCGGA

>*PtPhos3* upstream region, 1000 bp
CGCATTCGCAACCCGCTGGGTCGAATAAAGCGTTCGTATTTGGGAGCTTTCCGGGTAAAA
GAGTCTCCTACAAAGGTGCATTTGGTAACCACACGCTTCCACGCCTTGTTCTGTTTCTTAC
CGGTCCGCATGACTTTGAACATTTCGTCATCGGCTACCGGCTTGACCTTGGGAATGGGCA
CTTCCCATTTTCCGGCCTTTTCTTTCCGCTTTTGCTTAATCGTATTCGACAAAACCTTGGCA
CGGGAGACACCTTGACGGTCTAACAAATAGGCCGGTACGGCCCCATCGGCGACGGCATC
GTCGTCCGCGTGTTTGTTGTTGCGTTCGTTGTGCATCGCGATCGTTTTGCGCATGGTGGCC
TTTTCCTTGAACCGCTTCTTTTGGTAGAGTTTCGCCCGAATACCGTGTACTTTTTGGGCAA
ATTCGGAGCGCTTGTGCACCATACGCGCTTCCTTTTTGCGCTCACGCTCCTCGGCGTCGAA
CCGACGGCCGTGAAGCTTGGCGTGGCGTTCCATATGTTCGTGCTGCGGCATGGTGGAACG
AAGGACCTGTTGTTTATTGCGCAACTACGATTGTCGGAGAATATGAATGAATAAATGAAT
GAATAAATTGGTTGTACCGTCTCTCTTGTGCACAGACGCTATTGTCTAGCGATGCGAACA
AATCTGCCGAGATACGCCGGTGTACCAGCTTGGTTACTGGACGACGCGTCTATCCGGACG
AGAAAGCCCAGCGCGGAAAAACGGGAGATGCGCAAGTTTGGTCGGCATTATTTCCTTGGT
TGGCGAGTTGCCCACGGGACCGGATGACGTCCAAACCTAACGACACCGGAAAATGTCTG
ACATCTGTCCCAAAGCCGTGGACCTTATGTGAATTGTAAGACACACGCGCCCGAGAAGCA
AAACCGCACTCCGGCACGCGATTCGTCCTCCGGTGGGGGCGAATCTCAACGAACGGAGA
ATTTGACCGTACGCGTTCCGTCGTTCAATATTCTCATCGGTACACC

>*PtPhos3* downstream region, 538 bp
CACAATTCACATTTTTAAGTTTTCGAATGTGCTACAATAAACACCGTTTTTCTGGAGCTGT
TCCAGTTTTGTTCCTGTTGTTGGGATGCTCGGGCTGTAAGAGCTGCTTGATAGACGTTGAA
TATATACTGGTCGTCTAGTCCATTGGCCAGTACCTTGCTATAGAAGAATATCGGGTTATTG
CTTTCCAGAACCGCGGACACGTCGATTTGCATCATTGGAGGCTCCAAGCGCTTCCGATGG
TCCAAATTGCATTCGGATTGAAAGTGCCCGACCAAACCACAGTTCCAACAAGGTGTCGCC
GCTGCGGCAGCCTGTAAGAGGCAACTTTGAGCTTGCTTGTCGGGAGGAAGTGTGGGGCAC
TGTCGGAGCCAGTGGTCCTTCGAGTCACAAAGGAAGCAGACTCCGATTGATCGGTTCTTC
CTTCGCTTTGCTTCGTAGCGTTCATTGTTTTGCCTGCGTTGCCTACGCTTTTCGTCGTTTTC
GTAGCAGCAGACATACAAACAACGATCGGTAGGGGCAATATTCTTGGCCGTTA

>*PtPhos5* upstream region, 1073 bp
AGAAATCGCGTGGTTGGAAATTGATGCCCCCCGAGTTATCGTAGTACATTGTGTTGATGG
TACCGACGGCATGATCAAGTAGGGCGGCGTTGGCGGTGAGTCTTTCGTCCGGAGTCCCTT
CCCGCATAATGTTCCAGTAGCGCTCTTTAGAAGAAGGCAAACTGACCATTTCGCCTGCCA
ATACCGGTGCTATCATCACGGAACTAATTTGCAGTGCGACCAAAGCGCTGATGAGGCACT
TGGACGCGTATTGGGCAAACGTGGACCGGGAGTACGCGGGGGACGGCGGAGTGACCGCA
TCAGTGTTGGGAGCAAAGTCATCCGCATTGCACAAACGTTTCCGCGCCGTCAATGCCGTG
GTAGGACAAAGGCCTAGAAGTACCTTACTTGAACGTGAATCCAACGACGATCGTGTAGGT
GGTAAAATCCAAGCGTGCACGTCGCCTTGTGCAATAAAACCAATAGTGACGAGCAGCAG
TGGCGTGAGAAGCCGACGACCGGAGAAGTTCCGAATGTTCCGCAGGCTTTGGATCATCTC

ATTTCGAATCTCTCCTTGGGTGTACGGATACTCTGGGGGATTGTTACGGACTGGCAGTGGT
GCAATGTTTGATGGGTTTGACAGTGAATCGTCCCACAACCTTTCTGTTAGTATGCGACGAT
TGCACTCTATAGGAGTAACGGAAGGAAAGGGCGAGAGGGCAAATTCGGTAAACAGCTCT
TCGGTCCATGTAGGAATTCGGCAGAGAGCATGTCAGCCAACGTGTGCTGGTGGTTTTGCT
GACATCACGATTATACCAATGGTCCTATGGTATTCCACGGATTCCAAAGGATACTTTCCTT
CTTTATTCTCATTGGCCATAAATGTCGGACTTTGAACCTTCGCACCGTGCGATGCTCCTAG
CTAGTAGGACATATAGACTGGACACGTAGAGTAGTAGCACAGTATATCGGAAAGGAATA
CAAAGTGCATTGACCTGAATGGTCCTCTCACACAGTCAGTCTCTTGTATACGTACAAAGC
ACACACTCGGTTTCTAGTACCAGTGTATATGTGTGTATCGAACAAGAATACCA

>*PtPhos5* downstream region, 500 bp
GCTCCCGAATGTTTGTTTTCGTCTCCTAGAGAACAGGACAAGCTAGGCCTCCAGCACTCTC
CTTGGCACTGATTGGAGCGGAAACGATCACCGTATACAAACTGTAAATATGTGTTTGCTA
ATTGACAGAGTACACGTCTTGCCAATGCAAATTAAGCCAGCACTGAACACTTATTACCTT
AAAACATGGAACGCGTATTCGACTAGCCCGGAGTCGATTGAATTCAACCAACACGGAAC
ATTTCTTACGTCTACAGCGTGTTCGACTAGCCCGAAGTTGTTCGGTTTCCGTACTTTTCAA
TCACCTTTGCCAGCTCTTCCTTGGCCGAGTCCTGAGAAGAGAAAGAAGTCAGGTTTGTCA
AATTTACCAATACAAGGTTTAAGGATTTTTTTAATCACATACCTTGCAACTGTGACCCAGA
GTGCACTGTCCCAGAAGATTGTCTCGGGCAATGTCGATAATGTGGGATGTCAGATATTCG
TTCATCCACCGCACTTGC

>*PtPhos6* upstream region, 1000 bp
TGTGAAAGTTTCTGCTCAAGTAGCAAATTGTAGGACAATAGAAAGGCAAAAAGGGAAAA
CGAAAAGGATTTAAGAATAATGTGAGTAACGACTGAAAGGAAGCTGAGCTATTGTGCAA
CCTGTCGAATGAGTAATTGGTTGGCAAAAATGTCTTGCCATAGCTTTTAATCTAAAAAGG
TTTTGTGGCAACAAGTTCAAATTGTCCGCGGCTTCGAAATTGGTATCGAAAGTATCTGTCT
TTCGTATTTAGCAGTAAGCAGAGAAACATTCACCCAGAGCGAAGCACTCGAACAAAGTA
TACCTATGGATATAAATGCTACACTTGGAATACACTGCGTGCTCTCATACCTGAGGAATG
GAATGGCGATGTAGCTGCAAAAAAGTTGGTTCCGCCAACAGACTTTCGGAGAGCAATTGT
TTGTTTGCCTTCCTAACACACTCTTACAGTTAATCGACTGAGAAATAGAGATAATTTCCAT
TTTGCCGCGTTTAATTCGGGTCGTGCCCTTATTTTCCAAAGTCAAGATTCATCTCATTCTCA
AGATTCTTCGCAACATGACGTTTCTTGCTTCGTCGAGAATCTCTACATCGCATGAAAATGT
CTCTTTGTGAAGTCTTCCGTAGGAACTGGAACAAGCAATGCTGCCTTGGTGTAGGGCTCC
GTAAGGCTAGTAGTGGTAGTAGATTTGTTTGAACGAATTTGAAGTTTAGGTTGGCTGAGG
TCGCGAATCTCGGAGATGGATTGACAACTGCCACTGAGAACTCCCACGCCGCACGCACAA
CGATTTTTTCACAATACAGGCCTTGTTGCAACTGGCTAGAAAAGAAATAGACTCAGGCGT
ACTTGATGCACTGTATTCCATTTCGAATCCTCAAAAAAGAGATTTGGATTATCTGGTCCCT
TTTGTTGATTTCATTTTCCGATTATCCCTGTCTCGCTTTGCCAGACCCATTGAAACATATTC
GGGAAATCCACCCCCGCTTTTTCTTACAGTGAAC

>*PtPhos6* downstream region, 500 bp
AAAGTTTTTGTTTTTGTCCCTTTATAAAAATCATCGTTGGGACAAACGTTCTTCCCAATTA
TGAACCGGCTTGTAGTTTACAGTCGTAACATAGGCAGCGACGACGCGCCAGGTCCGTACC
ATGAAATCTAGAAGGCGCATTACGTATTCTTAACGTTAAAAAATGCTATTGACCGTAAGA
ACCCCTTGCCAGAATCTAAGACACGGCAGCAGCCTTGGCGTAGGTTACTTCGGGATTGGC
TCCGTCGATAGTCTTGGTCGACTGGGCGGGCGTTGGAGCACTCGCAGGCTCACCGTTTTC
CTTGACGAGTCCCAAGGGCTTGAGGACCGTCAAAAACAACGGTTTGAAAATCTCTTCCGT
TTTACCGACCGCCCCCATGACGGTCTGAACCACGGCCTTGATGGCAGGATTCAAAACGTT
ATCGTCCAAGCCGTGCAAGTTGTGCCTAAACGGAGGGGAATCGAGAGAACAGAAAGATA
CTTAGTATCATACGGTGTCG

>*PtPhos8* upstream region, 910 bp
ACAGGTTTGTTGCTTGGGAACAGCCGTCGCAACTGTCGTTGTGCTATAGTCTTCCGATGCC
TTTCTAGGCGTCAAAGGAGACTTTTAATTCTCGTGTAACAAGCAAGAATTGTTTCAAAAG
CCAACAGTACTTTTTCTTCTAATTTCACGTCTTGTACCGTTTCCCATCTTCATCGACAGTAA
GGTTGTTCGTCCCGTTGGCCTACGTAGTTTAGCTGTGAGCGCTGTCAGTTGCAGCTATCAC
AAGAAAAGACATTTGCACAGTGGCATTCATCATGCAAAAGACTGGTGGTGAATCAACAC
TTAATGTGCATTTGCTGTTCAAAACTCCTGGAGTCCTGGCGACAGTTTGTGCTCTGTATGC
TTGGGGTAAAGTGTTCACAGTCAACTGCTGTGAAGCAGATTGATCGAAGCTTGACGGGTT
GGAGGCAGCTCATCCATGCCAATGCGATACCTCCGCTATATCATACACAGAGCATACACC
AAGCATTGGATTATTTTCTAATTGCTAATTTTACTTAAAAGCCGTTTCGCGTCGAAATAGC
GATTTTTTCATGTCGCAGAGACCGAAATAGCGGTTTTCCTCAAGGTATTTCTATCCTTCTA
ATTGACGTTATGTATGACACATTTGCTGTAACACTGGGAGAGGTTAATTGAAAGTTCCCT
GCCAACTTATCTTACCAGATTCAAACTCTGAAAGGAGATTCATTCCTCCCACTCGCATTCC
AAACAAGATTCTTCTGCAAATCTTTTCGATTTGGAAAACAGATTCTTTTGATTCTTTCTGT
ACCATCTAGTATGCCTCATTTGTACCGACGCTCAAGTTCCTGCAGACCACAAAAAAGATC
TTTGCACATTTTCGTTTACAGTTTACTGTTACGCTGATTTCCCTGGTGAAAAGAATTCTTCG

>*PtPhos8* downstream region, 500 bp
TTTTGTGGAGTTCTTTTTCTTTATCAATCCGGATTGTGATTGTTTGAAGTTTCCCTGATCAA
TACCTGACATCGCTTGTCTACAACTACTGGACCGAAACGGATTCACAGTCACTGTCAGTA
CCTTTTGCATCTATATTTAACTACTCTTGATCGTTGGCTACGCCTGCAGAATTCAACTCTTC
ATACAAGTCTTTTACGCCGAATCTACATACGCACAAAATTGATCTGTATTAACAGAAAGG
CGGTCTACGGAACAACTATGTATGATACAAAAAGCATATTCTTCTCCCATAGAAGAACAC
TAAAATACGACTGCAAACCTTCTCATCATCAACTTATTCGACATCGTTGCGTTCGTCATCT
TCGAAGATGATCCGCTGTAGCTCACGCAAAATGGCATTCCCCAGGACTCGGTGTCCCTCC
ACCGAAAGATGAACATAGTCCGGCATAAGATCCGAAACAATCTGTTGCGACTTTGCCGTA
TAGTGGTCGTTGCCC

>*PtPho4* upstream region, 996 bp
GAAACACTGGTATTTAAGTCAAAGTCGTATCCACCGAGAACGCTACTAACTTGGACCTAG
TGCCAAACAATAGCTCTTTGGACGGGACGGGTGGCAATGCGGATACCAAGACTGCGTCC
ACAATCCAATACATCGCTGTGATCTTGTCGCCCTTGGTATCCTCCATGCCGTTACTGTCTC
CATCGGACCCTCCAGTGTCCCCTCGCTAATACCTTCCCGACTGCCTTCCACTCAAAAGTCC
TCATCACCTCCAGGTAGTTCCAGTGCCTTGTCATCGGGAGTGCCTTCGAGCGCAAAGTCG
GCTACGCTGTCGTCAAATCCTTCGATTGCACCGTTGTTGAGTGCGTTGGGTATTCCATTGG
CTTCACCGTCCTTGCAACCTTTGTCATTGCCGTCGGAAAGGCCGCCCAGTACGCCTTTGGA
AGCCCCGTCGGTATTTCCATATCCCTTTACGCTGACCCGAACCCTAATCTTCTGATGCCAC
ATTAAAAGAAAATTACACCGTTAAAAGTTTTGTGCGTAGGATACCAGTACTCGGAATCCG
CTTCGACGTCTTCGTATGCCATTGCGTAAAAATTGCACACTTCGAAGAGACCTCACGACA
GAGAGAAGGCAAACAAATCGACTTATGTTCGTTTTCTTTGCATCTTCGAGATTCATGCCG
AGGAGTCGCTTTTCTAGCGCTTCACTGTCATGCAACAGGTAAACTCGATTGATGGCTGCCT
GAGTCTTCCTCGAATTCTCTTTTCCAGCATGAAATCTTTGGCCGGAAATCGATTTTACCGG
TCTATTTGACTCCCATTACAATGATAGCCCTGAGTGAATTTTAAAGATCCGATACCGCAG
AGAGCTCTTGGATACAATGCGAACTGTTCCAGACATTGCGATGATTGAAAAGTGGAAAA
GTCGTCACCTGAAGGGGAGCTTATAAAACGACAGCCACTCTTCAACGATCTTCCCGAAAA
GCTAGCTTTCCATTTTGAGACCGATATCATC

>*PtPho4* downstream region, 500 bp
GACAGCTTGCGCATGCCCATGCACTGGATCCGTTTAAACAATGGGTAGAATATCCCAATT
TGAGTAGCACTTAAAATAATTCCATGAATTTATATGCATTCATAAATTGGAATAGTGCTTG
CTGTTAATTTCATGAATGACATATATGAAAAATAACTTGTATAACAGTGAATTCATAAAT
CAAAGAAAGAGAGGAAACTGTAAACTATTGGAAGGAGAAAGAGAATGCTGTTATAGCGA
AAGAGTTTGAAAGTAACAACAGGCTTATACCATCATTACTTTTGGTTTTCGTTTGTCGTCG
ACGTTTGTATCCTCCCTATCGTCATCATGATTGCCACTCTCGCGTCCGTCTTCTTGCCCTTC

TTCTGCAGCACCCGTCTCGGCATCATCATAAATTGCACAGCTCGTGTCTTCTTCGTTTTGA
CTCGTCGCGAGCTCCAGATGCGCTTCACCAGCTTCTTCCGGAAATCCTTCCCCTACCACAT
ACCCAGACTCGGTGT

>$PtHp_i1$ upstream region, 996 bp
TCAGACCTCGGGCAGCAAGCCTATAAGCCCTACTGGGGTTCAACGGTGGTTGATTATTTG
TTGAATCAGTCCAATGAATCTTCGTTGAGCATTATGGACGTTTCGAAAAGGACATCAATC
ATGGCCGAAGACATCGTTTTTACGTTGAATCAACTAGGGATTTTGAAGATCATCAACGGT
ATATACTTTATCGCAGCCGAAAAGAGCCTGCTTCAGCGATTGGCAGAAAAATACCCCGTA
AAGGAACCTCGAGTGGATCCATCCAAGCTTCATTGGACTCCCTTTTTGACTGACATCAAG
CGAGACAAGTTCAGTATACATAGCAAGAAGCCTAATGTTGAAACGGACGAAGTCCGAGG
TACGGGAGGCTTTTAAATGGTGGCTTTCCTCAACCAAATTGACGAAGCCAATATTACTTT
GAAAGGAGGTTTTCTACTGGCACACATTCGTTGGCTTGATGAGCCTTGTCTACACCTGGCT
AGGCAAGTTTGGTGCCACGCGTGTGTTCGTTTTTTGCCGCGTTGTTTCGCCTCTCATGAGA
AAGCTTACGATTGGGTAGAAAGTTGACTAATTGTATCACCAACAGTAAGGAATTCTGGCT
GTTGCGCTTGCCTTCACAAATACTTTCAAATCTTCTAGCAGACAATCATCTTTAACAGTTA
GTGCAACGTTGAGGCTACGCTGCTGCCTCTAGAGGATTCAAATACTGCGCAGAACCGTGG
GGCGGAAACAAAGTAGACTTTGATGGTTCAGGCAAAAACAGAGAATGCTCTCATCAGTG
AAAACATAACTCACCTTCGAGCTCCAAATAGTTGGCTGTCAAATTTCCGCCGGTTGACAA
TAGGTGTTCTCATTTTTTACGGACGCTCCTGTAATCATGATCGGACGGGAAAGCGAACTG
TCTCACTTTCATCAACTCACTATCAGTTCTGCGCCCACAAGTATTGGAAAAGCACCAACA
ATTCAGCAACTTACACTTGCATTAGCGTCGGTAAA

>$PtHp_i1$ downstream region, 500 bp
GAAGGCAACACAGTTATTAACTTTAAGGAAGAAAGAACTAGGTAGTTGATCACGAATCT
GTGGGTAAACAAAGAGCTTTACCGCGTACATTCACTTCAGTCTTCGCGGGCATATGTATA
GGAAACAAATAAGATTGTCTCCAATGCGGCAAAAATGAAGCTTGATTTGCACACACAGCT
AGAGCCTCTCTGATCCATCCACTCCAATTGATTGTAAAATATCACAGATGTACCATTTTAT
CTCGTTAGAAATACTCCTTTCCTTAAATACTCCATTACATGGTTCTTTCTATTTTTCGAATT
TTCTCGAGGAAAACCGCTATCCCGGTCTGCAAAACTTAAAAAATCGCTATTTCGATCAGA
TCTGAAAATCGCTAAGTATTTTTGAGTGAGCGCGAATCGGCTTTTAAGTAAAATTAACAA
TTATAATCCAATGCTCGGTGAATACTCTGCATATGATATAGCGGAGGTATGGCGCTGGCA
GGGATGAGCTGCTTCCAA

>$PtNap_i2$ upstream region, 991 bp
GGTACGATTTCGGGTGGACGCCAAAACGGACAAAATTTTGGGAGCCAGTATTGTGGGAG
TTGGCGCGGGAAATATGATTTCGGAAGTTACGCTTGCCATGCAGTCGGCAACTGGACTAG
GATCACTGGCCAATGTAATTCATCCGTACCCAACTACCGCTGAAGTCCTTCGCCAGTCTG
GCGATCTCTACAACCAAGACCAAATTGACAATGACGGCGAAAAAGATTCTCCGTGGTGTC
GTCAAGCTGCAACGTTAAAGGGACTTCTGTTACATGCATATTTCTTCGGGGAAAGTCGGA
CAAAGTGACTTTCTACATTTAAAGTCTGTGGGAATCAAGTTTTGGAGGTCAAAAGATCTC
ATAAATGTCAAAGATACTATCATGTTTGTTAGCAGAGACCACAAATATGAATCGCATACT
TCCAGAATTGCGAGCTGGATGACCATGATTCACCGTCAGGGGGGTATAGTGGTCAGTAGC
ATATTTCTGACCGTGAAGCAGTACCTGTACAATGTTTCTGGTGCAAACGTGATACTTACTC
GCATGGAGAGATAATGACGTAGAAGTTTCTTCTTTTTAGACAAGACCAGTACGCAGAGGG
TTGTTGACATCCCTCCTCAATGAAATTTCGTATATGTGGGCTCGTGGGGCACTGTCGATTG
AAGATACACTGACGTAAAAGTTTCATGACGTCGAAAGCGAAAATGAACTTTGACTTTGAA
GCTCCATGAATCTGGAAGATTCATGTTGACTTCTGTAGAATTGGTATATTTCTTACGTAGA
GATCTTTTGTCGATTATACCTTTGCGATGAGCGGAGGTGGCCTGGCTATGTTCCTTCATAG
TATCAATAGTGATGTCTCATTCCTTGCGAGCTCGAAATTTAAGAGATTTTTGGAGCTTGTT
TTGAAGGTCTGCTCACAATTTGCAGTGGCAGCGACCGTGAAACATTCTGACATACTGCGA
GCTCGAGCAATCGTTTGAAAATCAACC

>$PtNap_i2$ downstream region, 500 bp
GCATTCAATCACGAGAGGTCGATATCACCGTTGATCGGTTTGAGGTCCCCTCTATTTTCCC
ATATTTCTGTTTAGCCAAATAAATACTGTGCCGAGAGCTTTCTCGTTGTGTGTGGTTTTGC

GCACACTGCTGGAGAATATAACATCTTGGATTAAAGTGTCTTTTACTTCCCCTTTCTGTGC
TCCAACTTTAAATGGCTTGGTTGACTTCCTTGATCTCCAGCAGGATTAGTCCTTTTGAAAA
AATGGTGTTTGTTCAACCGGCGGCAACTACTACCTCTTTCTCCGGTTCACCGGATTGCCAT
CAAGTGTCTCGTTTTTCACAGCACATCATTCTGACGATCGCTTCCAAAGCCGACGTGAAA
AACGAGGAATATTGGTGAGTTGCTTCACTCTAGGCTGGAGAAACACGTTGGCCGGCGTCG
TCAAATCTTTCTTCCTATACACGATTGCAAAGTTCCATCTCACAATGCGGTATTCTTACTG
TAAATGCAATTTAT

>*PtNap*$_i$*4* upstream region, 1000 bp
AATTTGGTTGTTTTATTTGAATATTGTGCCAAGTCTGTTCTCGCTTGTTAAAGCTAGAGTT
ACTATAAACCTTTGGAATATACCACGCTTTTTGTTTGATTCCGTTGTACGTAGGTATTCTA
AGGTTCTGTGAAGCCCTCTTAAAACTAGGACTTTTGACCAGGGGGGAAATGGTCTACAAT
TAGAAACTCTATTCCAATTGGAAGAATCGCTCTCGCAAGAATCCTTCAATGAAACAAAAA
TGTTCACTGTCATTCTGGGAGGAGAGGAGGATGTGACCGTGTGCTACAAACTCAGTAGAA
GTAGGTTTTCACATTAAGACAAATATTCCATTTTTGATGTCCAAAGTCTCGGAGAATGGGT
CTTTTAAATCAGGGCCTGGAGCGGATTGATTGATACCACAGATTTGATCATTGCGAAACA
GAAAATTTCTCAAGTTTTCACTTCTCTAGTTTCTGATGCTTGTTCCTCACAGTCAGTATTAG
GCGAAACTTTATATTCTGGCAGTGAATTCCTTTCATTGTTGGCACGCTTGATCTATAGAGA
TTCGGACTAGAGTAAGAGAGTTTAGACTTGGGACGGATCATCTCGCTATCCTAAAAAATG
TACCTGAAAAATCAAAGTTTTTGGGACGATGTTGATTCACCCTAATGTAACATATGGATA
AATTTGAGTTGACGTTTATCGGAGACTTCACGCAGCTCATGTCAACAGTTCTGGACATGTT
TCTCTTTCTAAGAAAGAGATCGAACCGCGACTAAACGACGGAATTTTATATCGAAAGGAT
GACGTCAATCGGCGCTCCATTGCTTTTACAAAGAAAAAGATTTTCCGCGATGTGAATCTG
CGAGATTCGTGATCGATTCTGTAGAAACGGTATACTTCGTACGTAGATCTTGAAATTATCT
CGTGGATTTGTGTAGGAGGCCGTACATACGTGGCTCAGTCAGCTTGATGTGAGTTGTCAC
TTTCGCCTTTGGTCTGACTTTGAGGAGTCTCC

>*PtNap*$_i$*4* downstream region, 500 bp
GCACCAAACAAGAGGTCGATATCGCCGAATATTGGTTGGAGGTCCCCTTTTGATTATCCG
TTTAGTATATATAGTATATACAACCTGTGCCAAGACCATAGTTCTCGCAGATTCTGCTTGA
GAATAAAATAACTTGGGAATGTTTATCTTATTCTGACTGCATGTAGAAACATTATAGTATC
AGGATGCTGCACACAGCTCTTTTCATTTCAACTGCGGTTCAAACTAACTGTAAACCTTAAT
CGTTGTGCTGTCAATGGTCGGCGTCCCTAGCCGTAAAAAGGTTAACGCCACATTCATGAA
TCAATTGTTTCTTAACTTTGCTTCTGAATCTATCGGCGTGTACGTTTCTCCCTATTAAGGTC
TAAAGAGAGAATGTGACTTCGAGTAGAAATAGGATATCTATTCATATACGAACGATTTTC
ACTCACCTCGCCGTGAACTTCAACAATTATATCGACCCTGACAGCACGAGCAGTCGTGTT
GCACAGTGCACCCGA

>*PtVpt1* upstream region, 1025 bp
TTGGAAACCGAATTTTCAGCTGTGTGCGTACCCGCGATACTCTGATGTGCTGGAATAGCA
GCCGGGCTGCGTTGACTGGATTGGGCGTGGTATTCATCGTTCGTCATCGCTTGGCGTTCAC
TGTAGGCATTCTGCGAGTCAGGTTTTTTATCAGCGAGACCACCGCGGCACTCTACAACGG
TCTCTGAATTGTGTGGCTTATGCACAATGTCGACCAAAGAAGAAGTAGGGAAGGGGACA
GCCAACGAATCATTGGCTTTGACGCGAGTATTCACAACAGAGATTTTCGCGCCAACGTCG
ACTTTTTCCTTGTTTGAGGAGGAGCCAATACCGAGATTTCGCTGGTTCGTTGCCCGGGGAG
ACTGTACATTCATCCTCCGCTGCGAATGGGAAGGGCGCAACGACCCTTGGATGCTGTGGT
CGGTATTCATATGGTGACGGAAGTACTCAGTGCGTTGCCGAAACAAGCCAACGAAAATAT
TGTTGTATTCCAAATGGCGATTTTTGCTGTTTGTGCAGTCGCGTTGCTTACAGTGAAATAC
GAAATTAACAGTACCATAAAAAAGGAGGCAATGTGAGTTTTTCGTTCAAATTACCAAATT
CTTCCCAGTTCGACAGGATTTCCTGCGTTGGCAACAACATTCCTGATTCCATAGATTAAAT
TTCGAATCAAAGTATGGTAGGTATTGATAGACCAAAGCGAGATTCCATTTTGAATCTTAC
AGAAAAGTCTGTATCTCTGTCCACGCAAGGTTGTCTTTTTTGTTCGTGCTTTCGAAAGCAT
CGTTTCTGATATTGCGTGAAAAGCATAGGTATACCCATTTCATCGGCAAAACCGACTTTTT
TGCATCTATGGATTGTTGCAAAACACACTCGAATATATTGACTTACTGTTAGAATCTCGTA
GTGCAACTTTACGCATACGCAGAGGCGCTGCCGAGACGACGCGACGTAAACTCTAGTCGC
GTTCTTGAATTGTGAATGACGCCGTTACCGTTCCTTGGCGAAAGCTCAATTTTACAAAC

>*PtVpt1* downstream region, 503 bp
AGGTAGTAACATTGCCTCTTGGTGCTACTACACGATTTGATATCGATATCCTTCAGTACAT
GTTGACATGTGAGTAGAATGTGCAAATAAAAACGAAAACAATTTGCGGAGACTCTTCAA
AATATCATCCCCTTTAAAAATTTCCTTATTTTTATGGACACATAGTAAACTATATAGAAGT
TTGTGTTCTTATACATATACCTGCGGACTGGTGACTCACTTGTTCACTTTAGATCTGCTCC
GTTCATTGTTCCCTTCAACCCTTGAGCCTCCTTGTTTTGTAGCATAAATTCGTAGCCCCGCT
TTTTAATGTACGGGCGTAGAGATTTAGTATTGATTGGATTCCACCAACAGTGGGTGCAGC
GAATTATGGTATCCAAATTTTCTGGATCACAATGCATGCTGTGCTTGGATCGGTTGTCCCG
AATCGTGGATCTAATTTCGTCCTTGAGCTCCGTCGAAATGGTATGCTCTGACGCGTACAT
GTGAGTCACCTTGGTATC
